# Behavioral and biochemical changes associated with the analgesic effects of (2R,6R)-hydroxynorketamine alone and in combination with meloxicam following disk puncture in mice

**DOI:** 10.3389/fpain.2025.1574474

**Published:** 2025-06-12

**Authors:** Vaskar Das, Isabella Milejczyk, Michael B. Basovich, Mario Moric, Jay Kaila, Craig J. Thomas, Asokumar Buvanendran, Robert J. McCarthy

**Affiliations:** ^1^ Department of Anesthesiology, Rush University Medical Center, Chicago, IL, United States,; ^2^ Division of Preclinical Innovation, Chemistry Technologies, National Center for Advancing Translational Sciences, National Institutes of Health, Rockville, MD, United States,

**Keywords:** (2R, 6R)-hydroxynorketamine, lumbar disk puncture, musculoskeletal pain, allodynia, non-opioid analgesic, glutamate receptor ionotropic (GluA1, GluA2), brain derived neurotrophic factor (BDNF), c-Fos

## Abstract

**Introduction:**

Low back pain affects around 619 million people globally and is the most prevalent musculoskeletal condition worldwide. Low back pain is often difficult to treat with traditional drug combinations, and opioids are prescribed for up to 60% of patients with debilitating low back pain. This study aimed at characterizing the analgesic effect of (2R,6R)-Hydroxynorketamine, an α-amino-3-hydroxy-5-methyl-4-isoxazolepropionic acid receptor dependent analgesic agent, alone or in combination with meloxicam in a murine lumbar disk puncture model.

**Methods:**

Male and female C57BL/6J mice underwent lumbar disk puncture and developed tactile allodynia. At day 7 postoperatively, mice were randomized to receive intraperitoneal saline, (2R,6R)-Hydroxynorketamine, meloxicam or both drugs co-administered for 3 consecutive days. Analgesia was assessed at baseline and 24 h following each injection using von Frey testing of both hind limbs and the area under the paw withdrawal curve (AUC_0−3d_) was determined. Brain, spinal cord, and dorsal root ganglion tissues were obtained for immunohistochemistry and western blot analysis.

**Results:**

Prior to disk puncture paw withdrawal thresholds were 3.44 ± 0.51 g before surgery and were reduced to 0.54 ± 0.38 g at day 7 without a difference by sex; however, sex-specific responses were evident in other behavioral outcomes. EC_50_ estimates for (2R,6R)-Hydroxynorketamine were 14.2 mg/kg (95% CI: 10.3 mg/kg to 19.7 mg/kg) in male and 16.9 mg/kg (95% CI: 12.8 mg/kg to 22.3 mg/kg) in female mice (*P* < 0.637). (2R,6R)-Hydroxynorketamine plus meloxicam enhanced the analgesic effect on the AUC_0−3d_ of meloxicam alone. (2R,6R)-Hydroxynorketamine analgesia was associated with increases in Glutamate receptor A1 & A2, p-Kv2.1, p-CaMKII and reduced BDNF protein ratios in the hippocampus, attenuated c-Fos in the spinal cord, and decreased BDNF at the dorsal root ganglion (DRG).

**Discussion:**

Our findings demonstrated that the analgesic benefit of (2R,6R)-Hydroxynorketamine is dose dependent, protein analysis suggests that (2R,6R)-HNK analgesic is associated with augmenting GluA1, GluA2, CaMKII, Kv2.1 and a reduction in BDNF protein ratios in hippocampus, decreased spinal cord c-Fos and reduced BNDF at the dorsal root ganglion. (2R,6R)-Hydroxynorketamine also augmented meloxicam analgesia in disk puncture mice. Our finding supports further study of the clinical potential of (2R,6R)-Hydroxynorketamine as a non-opioid analgesic for discogenic back pain.

## Introduction

Low back pain (LBP) affects nearly 10%–30% of Americans each year and remains a leading cause of disability globally (1). LBP is often difficult to treat, requiring a multidisciplinary approach including pharmacological, psychological, physical therapy, and interventional approaches (2). Acetaminophen and nonsteroidal anti-inflammatory drugs (NSAIDs) are widely accepted as a first line of treatment for LBP (3). Acetaminophen's high safety profile and affordability makes it a favorable option, although it lacks anti-inflammatory effects (4). NSAIDs provide a more effective analgesic option; however, they are associated with gastrointestinal, renal, and vascular adverse effects and exhibit a ceiling effect which limits dosing (5). These agents are often combined with gabapentinoids or a tricyclic antidepressant such as amitriptyline when neuropathy is present (6). Despite these drug combinations inadequate analgesia frequently persists, and opioids are often prescribed for patients with debilitating pain (7). While opioids present an effective pain management strategy for limited use, the risk of dependency and significant number of side effects hampers their clinical utility for long term administration (8).

Ketamine has been demonstrated to reduce opioid consumption in patients suffering from low back pain (9, 10, 11). Limitations to ketamine use include sympathetic stimulation, psychomimetic disturbances, abuse potential and hepatobiliary toxicity with long term use (12). Ketamine is rapidly metabolized into over twenty characterized metabolites including the active metabolite (2R,6R)-Hydroxynorketamine (HNK) (13). Preclinical studies from our laboratory have shown that (2R,6R)-HNK demonstrates analgesic effects in multiple murine pain models (plantar incision, spared nerve injury, and tibial fracture) (14, 15). Yost et al, replicated the analgesia effect we observed in the spared nerve injury model, and demonstrated that (2R,6R)-HNK analgesia was blocked by a α-amino-3-hydroxy-5-methyl-4-isoxazolepropionic acid (AMPA) receptor antagonist and not the opioid receptor antagonist naloxone (16). This suggests that (2R,6R)-HNK may represent a non-opioid drug with potential for future therapeutic studies for pain patients.

The purpose of this study is to evaluate the anti-allodynic dose response of (2R,6R)-HNK by as well as the combined analgesic efficacy of (2R,6R)-HNK with meloxicam in a preclinical disc puncture (DP) model of low back pain. We chose this model because both inflammatory as well as neuronal changes are involved in the evolution and duration of hypersensitivity (17), and then disk puncture model has been shown to have similar hallmarks of disk degeneration in humans (18). We will also build upon our prior findings of protein changes in the hippocampus associated with (2R,6R)-HNK by evaluating protein changes associated with pain pathways at both the level of the spinal cord and dorsal root ganglion (DRG). These studies should better elucidate if the analgesic activity of (2R,6R)-HNK analgesic efficacy is associated with changes in central or peripheral pain processes or both.

## Materials and methods

### Animals and baseline testing

Animal experimental procedures and protocols were approved by the Institutional Animal Use and Care Committee of Rush University Medical Center (IACUC Protocol IDs: 18-047, 18-063; 20-078; 23-044) and adhered to the Animal Research: Reporting of *in vivo* Experiments (ARRIVE) guidelines and the Guide for Care and Use of Laboratory Animals. The drugs, reagents, supplies, and equipment used in these studies with identifier and suppliers is listed in Supplementary Table S1. Antibodies for western blot and immunohistochemistry studies, suppliers and Research Resource Identifiers (RRIDs) are listed in Supplementary Table S2.

Male and female C57BL/6J mice approximately 9–12 weeks age, 15–22 g body weight were obtained from The Jackson Laboratory. We chose C57BL/6 mice for the lumbar disc puncture model study, as this strain had been used in our prior study describing the pain characteristics of the model (19). Mice were housed 4–5 animals per cage, fed a Teklad Global 18% Protein Diet (Envigo, Madison, WI) with food and water available *ad libitum* with 12-h day and night cycles.

Although aggregated numbers of animals are reported in the methods and results section of this manuscript, surgeries and behavioral testing performed in these studies were performed separately in male and female mice. The surgical and testing laboratory was cleaned and disinfected prior to studies of the opposite sex. Male and female animals used in these studies were not housed in the vivarium at the same time with at least two weeks between the surgeries or testing sessions of animals of either sex.

Prior to surgery mice were weighed and tested for mechanical allodynia by assessing paw withdrawal responses to stimulation with calibrated von Frey nylon filaments (0.02–6.00 g) using an iterative up-down method while standing on a grid (20, 21). Hind paw withdrawal responses are very reproducible and can be used to assess effectiveness in chronic pain states over a long period of time. The allodynic response to lumbar disk puncture in C57BL/6 mice is evident on day 7 post surgery and persists for more than 40 days (19). Lumbar disk puncture produces allodynia bilaterally, so the average value of both hind paws was taken as the response. A non-allodynic C57BL/6J mouse in the age range used in this study has a withdrawal threshold (PWT) between 3 and 4 g, and a response less than 1.3 g was considered to represent allodynia.

### Lumber disc puncture surgery

Mice (*n* = 144) underwent lumbar disc puncture surgery as previously described (19). The L4–L5 discs were then exposed and a 25G hypodermic needle, with a stop limiting depth penetration to 0.5 mm, was used to make a single puncture in each disc. The nucleus pulposus gelatinous tissue was seen inside the needle lumen upon removal. The skin incision was closed with 4-0 nylon suture.

### Dose response of (2R,6R)-HNK in lumbar disc puncture mice

PWT testing was repeated on postoperative day 7 and mice were then randomly allocated into 6 treatment groups: (2R,6R)-HNK 1 mg/kg (*n* = 18), 3 mg/kg (*n* = 18), 10 mg/kg (*n* = 18), 20 mg/kg (*n* = 18), 30 mg/kg (*n* = 18), and saline (*n* = 20). (2R,6R)-HNK or saline was administered via an intraperitoneal injection (IP) daily for 3 consecutive days. Allodynia testing was performed 23 h after drug injection.

### Pharmacological blockade with AMPA and opioid antagonist

Mice from the saline group were reserved for histological evaluation (Section 2.5). Repeat PWT testing was performed 7 days later the animals from the other groups. Mice exhibiting allodynia (*n* = 36) were given saline followed 10 min later by (2R,6R)-HNK 20 mg/kg IP for 3 days with allodynia testing 23 h after each drug injection. Seven days later the mice were randomly allocated into 2 groups stratified by sex. Group 1 received naloxone 1 mg/kg (*n* = 18) (subcutaneously) and group 2 received the brain penetrating AMPA antagonist (1,2,3,4-Tetrahydro-6-nitro-2,3-dioxobenzo [f]quinoxaline-7-sulfonamide (NBQX) 10 mg/kg (IP) (*n* = 18). Ten minutes later both groups received (2R,6R)-HNK 20 mg/kg IP. Injections were administered for 3 days with von Frey testing 23 h after each drug injection. Injections volumes were 0.05 ml.

### Co-administration of (2R,6R)-hydroxynorketamine with meloxicam

Co-Administration of (2R,6R)-HNK with meloxicam studies were performed on (*n* = 34) C57BL/6J lumber disc puncture mice. Mechanical allodynia was assessed starting on day 7. Mice were allocated into 2 groups stratified by sex. Group 1 received saline, and group 2 received meloxicam 10 mg/kg IP twice a day for 3 consecutive days with testing performed 24 h after the injections. Six days later mice were again tested and then allocated int 2 groups stratified by sex. Group 1 received meloxicam 10 mg/kg BID and (2R,6R)-HNK 10 mg/kg daily, and group 2 received meloxicam 10 mg/kg BID and (2R,6R)-HNK 20 mg/kg daily. The drugs were injected IP for 3 consecutive days with von Frey testing 23 h after each injection.

### Biochemical analysis

#### Harvesting of tissue

Saline treated disk puncture mice (*n* = 20) from the dose response study were used for protein analysis. Mice were administered saline or 20 mg/kg (2R,6R)-HNK IP for 2 consecutive days and then euthanized using carbon dioxide inhalation. Upon loss of neuronal reflexes mice were decapitated, hippocampi were dissected out and left and right hippocampus were stored separately in cold (4 °C) Syn-Per reagent. Dorsal root ganglion (DRG) tissue was isolated from each mouse and stored in cold (4 °C) Syn-Per reagent, and isolated spinal cord were stored in cold 4% paraformaldehydefor 7 days.

#### Immunohistochemical study of spinal cord protein modulation

Preparation of spinal cord tissue samples and staining for immunohistochemical analysis is described in Supplementary Text S1. In addition to the samples from saline and (2R,6R)-HNK treated animals, 6 spinal cord samples from sham animals were included in the in the immunohistochemical analysis.

Sections were incubated overnight at 4 °C with specific primary antibodies diluted in 1% NGS against protein C-Fos and neuronal nuclei marker (NeuN). Sections were incubated with the appropriate secondary antibody (diluted in PBS) for 1–2 h at room temperature in the dark. Secondary antibodies included Alexa Fluor 546 goat anti–rabbit immunoglobulin G (IgG) (H + L) or Alexa Fluor 555 rabbit anti–mouse immunoglobulin G (IgG) (H + L); Alexa Fluor 488 goat anti–mouse IgG (H + L) or Alexa Fluor 488 goat anti–rabbit IgG (H + L). Secondary antibodies were diluted 1:250.

The sections were washed with PBS and incubated for nuclear counterstaining with 4′,6-diamidino-2-phenylindole; 1:10,000 dilution for 10 min. Following additional washes, coverslips were placed with Fluoromount™ aqueous mounting medium and left to dry at room temperature in the dark. Immunofluorescence was observed on a Nikon Eclipse E600 fluorescence microscope and images were captured using a 2.3 MP camera. Immunoreactive neurons were counted using ImageJ software (version 1.53).

#### Western blotting studies

Protein preparation, gel preparation and gel loading for western blot assays are described in Supplementary Text S1. Protein (22.5 µg) was loaded into each well of 15 well plate containing a house-made 10% gel. The first well was used for the protein ladder and the remaining 14 were used for sample analysis, 7 for (2R,6R)-HNK and 7 for saline. Proteins were separated by electrophoresis with a running buffer using a stepwise increase method (30 V: 15 min, 50 V: 10 min, 70 V: 5 min, 90 V: 60 min). Protein was transferred to a PVDF membrane using a semi-dry transfer method and was then washed with buffer, blocked with 2% bovine serum albumin, incubated with the primary antibody overnight, and then incubated with horseradish peroxidase secondary antibody for 2 h. Gels were imaged with chemiluminescent HRP substrate and ECL chemiluminescent substrate at a mixture of 1:2.

Ratios of the following proteins to house-keeping protein GAPDH were determined using a Bio-Rad Gel Doc system. Hippocampal samples were evaluated for the expression of glutamate receptor 1 (GluA1), glutamate receptor 2 (GluA2), brain derived neurotrophic factor (BDNF), phosphorylated calcium calmodulin-dependent protein kinase II (p-CaMKII), phosphorylated voltage gated potassium channel 2.1 (p-Kv2.1) and glyceraldehyde 3-phosphate dehydrogenase (GAPDH). Additional proteins assessed from hippocampal tissue included: phosphorylated protein kinase B (p-AKT), phosphorylated extracellular signal regulated kinase 1 and 2 [p-ERK(1/2)], CXC chemokine receptor 4 (CXCR4), phosphorylated eukaryotic translation initiation factor 2 subunit 1 (p-EIF2SI), and phosphorylated eukaryotic translation initiation factor 4e (p-EIF4E). DRG samples were evaluated for transient receptor potential ankyrin 1 (TRPA1), tyrosine protein kinase B (TrkB), p-ERK(1/2), CXCR4, BDNF, and p-EIF4E and GAPDH. Ratios of the protein to GAPDH in the same lane of the gel were quantified using ImageJ software.

### Statistical analysis

The distribution of the primary and secondary outcomes was evaluated using the Shapiro–Wilks test and examined graphically using q–q plots.

The primary outcome of the dose response study (Section 2.2) was the antiallodynic response calculated from the PWT*day curve over the three days of injections (AUC_0–3d_). The AUC_0–3d_ was calculated using trapezoidal integration and the allodynic response for the 1, 3, 10, 20 and 30 mg/kg (2R,6R)-HNK assuming the saline group represented a 0% response and pre-surgical PWT extrapolated to 3 days represented a 100% response. The antiallodynic responses were then fitted using a four-parameter log-logistic equation: Response(log(*x*)) = Max + (Min − Max)/(1 + (log(*x*)/Inflection point)^Hill slope^). Max and Min represent the maximum and minimum response values, the inflection point is the response where (max − min)/2, the Hill Slope is the steepness of the curve around the inflection point. Confidence intervals of the EC_50_ were calculated using the likelihood method. Male and female animals were fitted separately and the difference of the individual EC_50_ estimate with the common estimate was compared using analysis of variance. The number of animals per dose (*n* = 17) for the EC_50_ estimation was determined from prior studies on the increase in von Frey force threshold with (2R,6R)-HNK (15, 16). In that study the average effect size (Cohen D) in the disc puncture models after 3 daily injections of (2R,6R)-HNK 10 mg/kg was 1.69 (95% CI: 1.38–2.01), 20 mg/kg was 2.13 (95% CI: 1.82–2.44) and 30 mg/kg 2.37 (95% CI: 2.07–2.66). Assuming an even effect across the six study groups (*f* = 42.1 and an *f* = 1.62 for paired comparisons), average sample sizes of 17 per group achieves 80% power to detect difference among the means at an alpha of 0.05 using a one-way ANOVA.

Secondary outcomes of the behavioral studies were the sex difference in animal weights, PWT's at baseline and at day 7 following surgery (Section 2.1) and antagonism of the (2R,6R)-HNK antiallodynic effect by naloxone or NBQX (Section 2.3). Weights were compared using an independent sample *t*-test. PWT's were compared using generalized estimating equations (GEE) with mouse id as a subject variable and time of assessment as the within subject variable. The link function was gaussian and the working correlation matrix structure was exchangeable. Time of assessment, and sex were used as factors in the model and weight as a covariate. The number of mice needed to assess a sex difference in PWT's at baseline and day 7 following surgery was determined from our prior study demonstrating a gender difference in the murine discogenic pain model to be 20 to provide a power of 0.80 at an alpha of 0.05 using a 2-sample *z*-test at a Cohen effect size of 0.93 (15). To evaluate the pharmacological antagonism of the 2R,6R-HNK allodynic effect, AUC_0−3d_ were compared among the saline, naloxone and NBQX groups using a GEE as above. Pairwise comparisons were adjusted for multiple comparisons using the Bonferroni method.

The primary outcome of the experiments on co-administration of meloxicam and (2R,6R)-HNK was the AUC_0−3d_. Data from the dose response study for (2R,6R)-HNK 10, 20 and 30 mg/kg were also used in the analysis. AUC_0−3d_ were compared using a GEE with mouse ID as the subject variable and injection number as a within subject variable. The link function was gaussian and the working correlation matrix structure was exchangeable. Group and sex were used as factors in the model and weight as a covariate. Pairwise comparisons among the group were adjusted for multiple compassions using the Bonferroni method.

The variable of interest in the immunoblot studies was the ratio of the average optical density units of the target protein relative to housekeeping protein (GADPH). Protein ratios were compared between saline and (2R,6R)-HNK using a generalized linear model (GLM) with sex and group as factors. *post hoc* comparisons were corrected for multiple comparisons using the Bonferroni method. The number of animals for the immunoblot studies (*n* = 20 per group) was selected to allow for sufficient tissue to perform immunoblot assays in triplicate. Assessment of the c-Fos co-localized in spinal cord with NeuN neurons marker activity spinal cord was determined as the differences in the RGB ratio of neurons labeled for c-Fos between sham, saline and (2R,6R)-HNK mice compared using a one-way analysis of variance.

Data were analyzed using RStudio version 2024.09.0 Build 375 (Posit Software, PBC, Boston, MA; URL: http://www.posit.co/) and R version 4.4.2, release date October 31, 2024 (The R Foundation for Statistical Computing, Vienna, Austria).

## Results

Mean male mouse body weight on arrival was (19.8 ± 0.10 g) and female was (18.7 ± 0.16 g), difference 1.07 g (95% CI: 0.61–1.52 g, *P* < 0.0001). Prior to surgery, mean (95% CI) weight adjusted PWT's were 3.50 g (3.39 g, 3.60 g) in male and 3.37 g (3.24 g, 3.48 g) in female mice, difference −0.13 g (95% CI: −0.08 g to 0.35 g, *P* = 0.590). Weight adjusted mean PWT's at day 7 were 0.62 g (0.51 g, 0.72 g) for male and 0.44 g (0.31 g, 0.56 g) for female mice, difference −0.17 (95% CI: −0.39 to 0.05) g, *P* = 0.223 (Supplementary Figure S1).

### Dose dependency of (2R,6R)-HNK anti-allodynia in lumbar disc puncture mice

**Figure 1:**
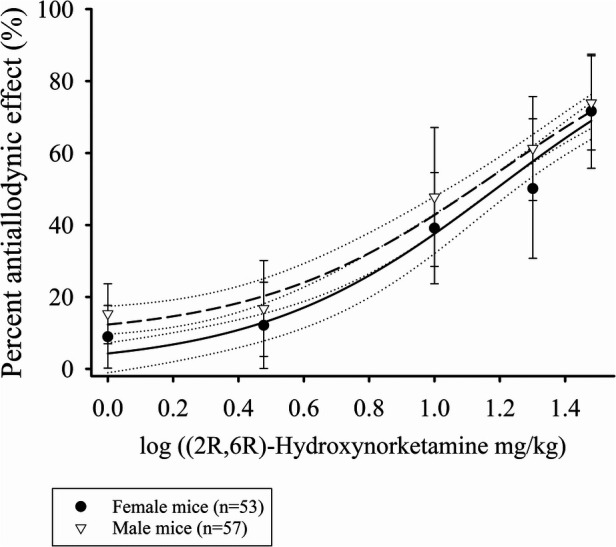
Dose response curves of the percent antiallodynic effect of intraperitoneal (2R,6R)-Hydroxynorketamine in a murine disk puncture model of low back pain. Closed circles are mean ± SD responses in female and open inverted triangles the mean ± SD in male mice. Solid line is the best fit line for female (*r*^2^ = 0.783) and short dashed the best fit line for the males (*r*^2^ = 0.793). The dotted lines represent the 95% confidence limits of the best fit lines.

The antiallodynic effect of increasing doses of (2R,6R)-HNK in male and female mice is shown in Figure 1. EC_50_ estimates for (2R,6R)-HNK were 14.2 mg/kg (95% CI: 10.3 mg/kg to 19.7 mg/kg) in male and 16.9 mg/kg (95% CI: 12.8 mg/kg to 22.3 mg/kg) in female mice (*P* < 0.637). The combined EC_50_ for male and female mice was 15.5 mg/kg (95% CI: 12.6 mg/kg to 19.2 mg/kg), the Hill slope 1.2 (95% CI: 0.8–1.5) and the *r*^2^ = 0.789. Individual mouse responses (AUC_0−3d_ g*d) to (2R,6R)-HNK are shown in Supplementary Figure S2, and daily PWT responses are shown in Supplementary Figure S3.

### Pharmacological antagonist studies

**Figure 2:**
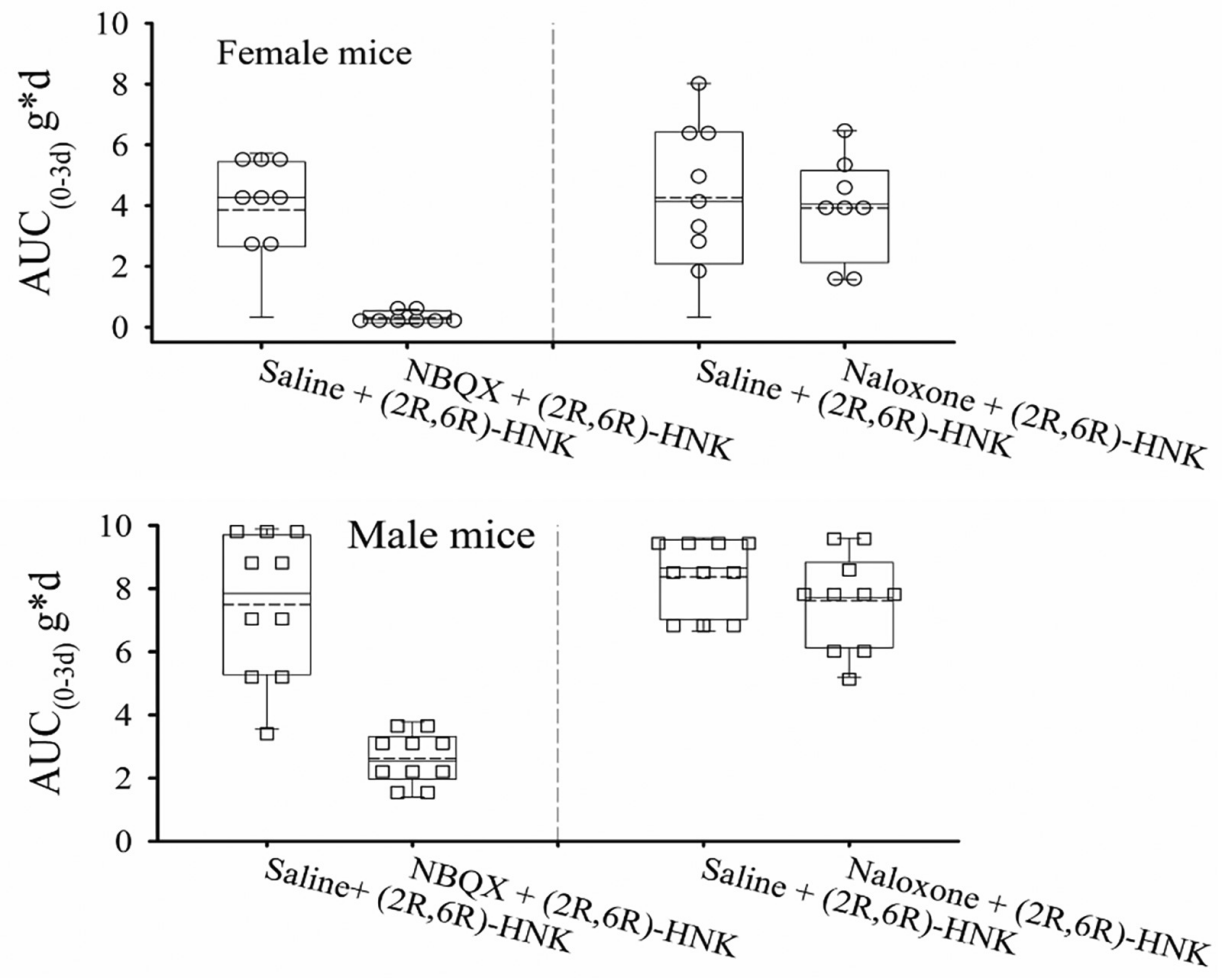
Dot and box plots of AUC_0−3d_ of the effect of pretreatment with naloxone and NBQX on the antiallodynic efficacy of (2R,6R)-HNK in disk puncture mice. The solid line is the median, the dashed line is the mean, the box represents the 25th to 75th percentiles, the whiskers the 10th and 90th percentiles. Combined male and female estimated marginal mean (95% CI) AUC_0−3d_ values adjusted for weight for saline, naloxone and NBQX pretreatment following by (2R,6R)-HNK 20 mg/kg were: 6.19 (4.01, 6.17) g*d, 6.03 (5.34, 6.72) g*d, and 1.10 (0.39 g*d, 1.81) g*d, respectively. Upper panel female mice: AUC_0−3d_ decreased with NBQX pretreatment compared with saline pretreatment, difference −3.86 g*d (99%CI −5.70 g*d to −1.99 g*d, *P* < 0.0001), but not with naloxone pretreatment, difference 0.10 g*d (95% CI: −1.75 g*d to 1.96 g*d) *P* = 1.00. Lower panel male mice: AUC_0−3d_ decreased with NBQX pretreatment compared with saline pretreatment, difference −6.34 g*d (99% CI −8.18 g*d to −4.49 g*d, *P* < 0.0001), but not with naloxone pretreatment, difference 0.22 g*d (95% CI: −1.43 g*d to 1.88 g*p, *P* = 1.00).

Seven days following the dose response study, female mice demonstrated lower PWT's 0.15 ± 0.17 g compared with male 0.72 ± 0.35 g difference −0.57 g (95% CI: 0.39 g to 0.76 g) *P* < 0.001. Female mice also demonstrated reduced AUC_0−3d_ compared with males following saline + (2R,6R)-HNK administration for 3 days, difference −2.7 g*d (95% CI: 0.78 g*d to 4.63 g*d). *P* = 0.001 (Figure 2). NBQX blocked the antiallodynic effect compared to saline, difference 5.09 g*d (95% CI: 4.01 g*d to 6.17 g*d, *P* < 0.001), but naloxone did not decrease the antiallodynic effect compared with saline, difference 0.16 g*d (−0.85 g*d to 1.18 g*d, *P* *=* 1.00). Daily mouse PWT responses stratified by sex are shown in Supplementary Figure S4.

### Co-administration of (2R,6R)-HNK with meloxicam

**Figure 3:**
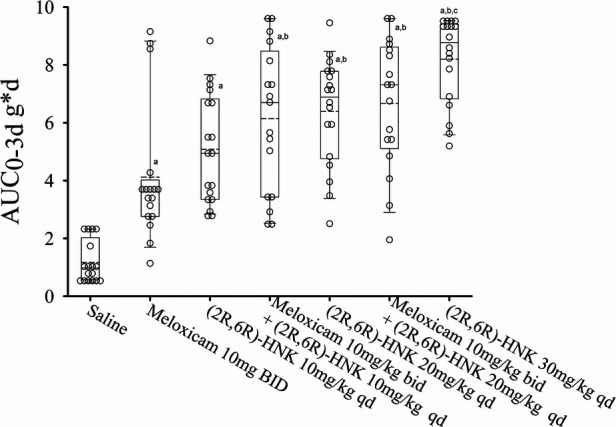
Dot and box plots of the AUC_0−3d_ for disc puncture mice receiving saline, meloxicam, (2R,6R)-HNK of combinations of meloxicam and (2R,6R)-HNK. The solid line is the median, the dashed line is the mean, the box represents the 25th to 75th percentiles, the whiskers the 10th and 90th percentiles and the solid circle the 5th and 95th percentiles. There was no drug by sex effect on the AUC_0−3d_ (*P* = 0.760) response. a = different from saline, *P* < 0.05. b = different from meloxicam, *P* < 0.05. c = different from (2R,6R)-HNK 10 mg/kg, *P* < 0.05.

Combined sex responses for the meloxicam alone or in combination with (2R,6R)-HNK are shown in Figure 3. Meloxicam 10 mg IP bid increased the AUC_0−3d_ compared with saline, difference 2.97 g*d (95% CI: 1.05 g*d to 4.88 g*d, *P* < 0.001). Meloxicam plus (2R,6R)-HNK 10 mg/kg and 20 mg/kg increased the AUC_0−3d_ compared to meloxicam, difference 1.99 g*d (95% CI: 0.43–4.21, *P* = 0.012) and 2.53 g*d (95% CI: 0.80–4.26, *P* = 0.005), respectively. The AUC_0−3d_ for (2R,6R)-HNK 10 mg/kg was not different from meloxicam, difference 0.98 g*d (95% CI: −0.79 to 2.76, *P* = 0.575) but was less than that produced by (2R,6R)-HNK 30 mg/kg, difference −3.16 g*d (95% CI: −1.22 to −5.10, *P* < 0.001); however, the combination of meloxicam with (2R,6R)-HNK 10 mg/kg or 20 mg was not lower than the AUC_0−3d_ produced by (2R,6R)-HNK 30 mg/kg, difference −2.12 g*d (95% CI: −0.26 to 4.52, *P* = 0.144 and −1.59 g*d (95% CI: −0.79 to 3.97, *P* = 0.879), respectively. There was no sex by dose effect *P* = 0.957. Daily mouse PWT responses stratified by sex are shown in Supplementary Figure S5.

### Effect of (2R,6R)-HNK administration on spinal cord C-fos expression

**Figure 4:**
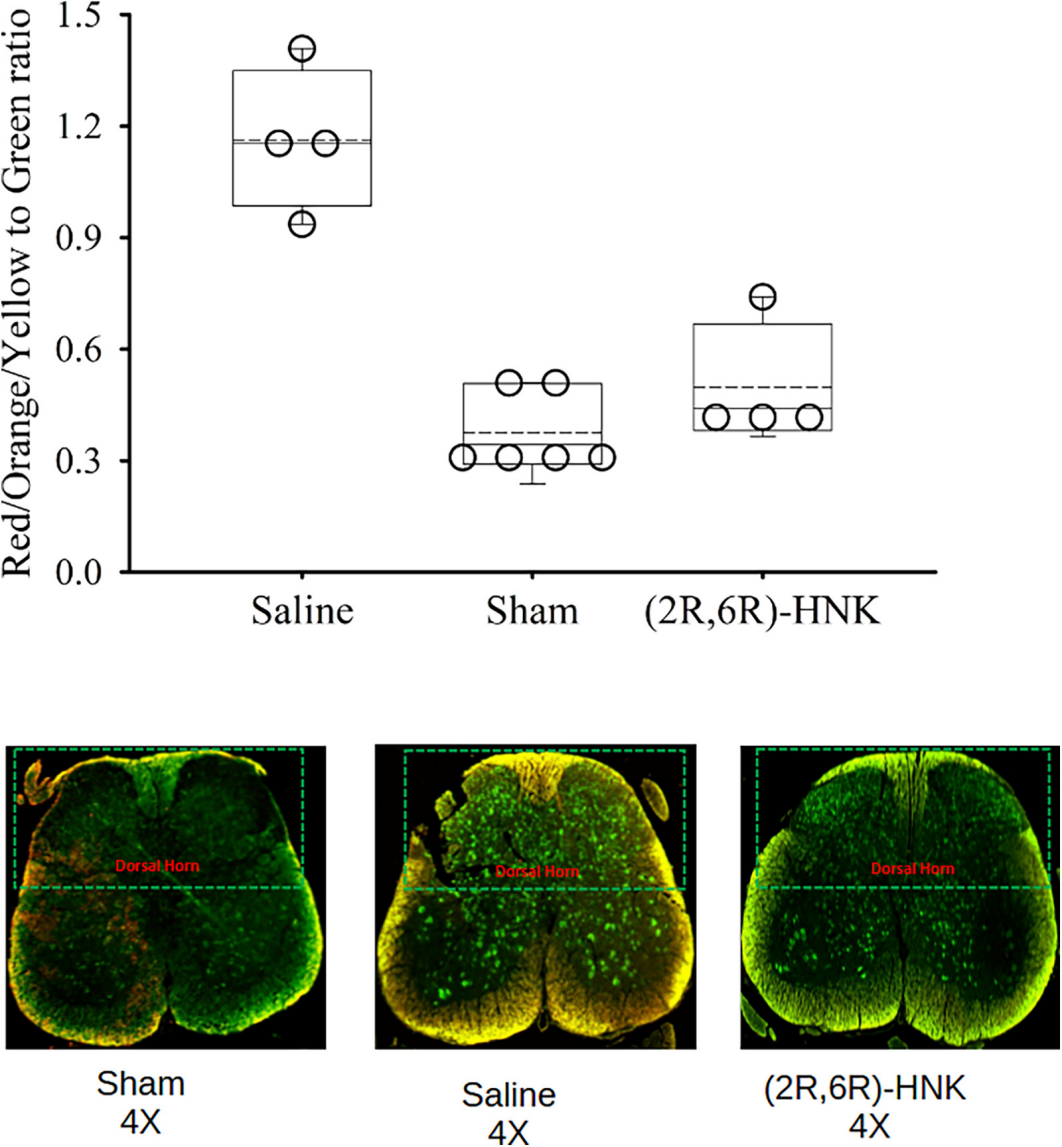
Dot and box plots of the red/yellow/orange to green ratios of spinal cord neurons labeled for c-Fos among saline, sham and (2R,6R)-HNK treated mice. The dorsal horn is oriented to the top of the figure. The ratio for c-Fos staining was 1.16 (95% CI: 0.85–1.47) in saline treated animals, compared with 0.50 (95% CI: 0.23–0.76) in (2R,6R)-HNK treated animals and 0.38 (95% CI: 0.26–0.49) in sham controls. The difference in the ratios between saline and (2R,6R)-HNK was 0.67 (95% CI: 0.35–0.98, *P* *=* 0.002) and the difference in rations between sham and (2R,6R)-HNK was −0.12 (95% CI: −0.329 to 0.08, *P* = 0.20).

The results of the immunohistochemistry assessment for C-fos labeled spinal cord neurons is shown in Figure 4. Saline treated mice have a greater RGB ratio of neurons with c-fos staining and was upregulated in the spinal cord of DP mice compared with the (2R,6R)-HNK and sham control group (*P* < 0.001) (Figure 4).

### Effect of (2R,6R)-HNK administration on protein expression in the hippocampus

**Figure 5:**
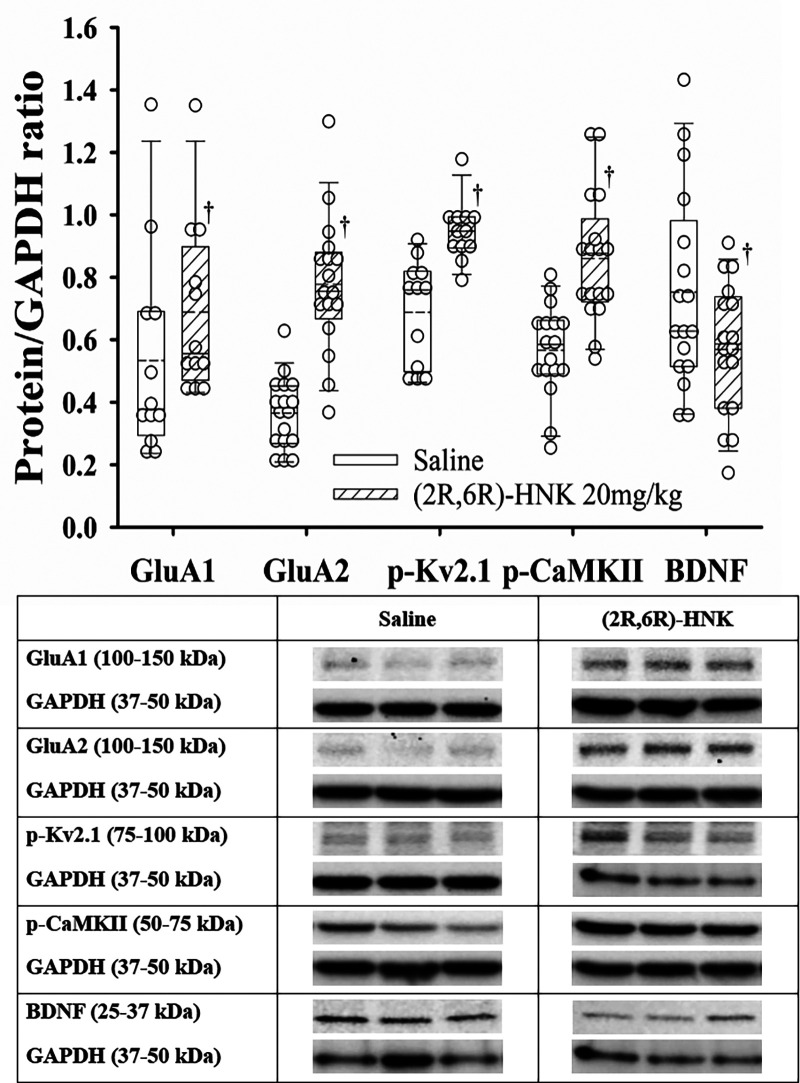
Dot and box plots of hippocampal protein/GAPDH ratios for saline and (2R,6R)-HNK treated animals following disk puncture surgery. The solid line is the median, the dashed line is the mean, the box represents the 25th to 75th percentiles, the whiskers represent the 10th and 90th percentiles, and the solid circle represents the 5th and 95th percentiles. † =(2R,6R)-HNK different from saline vehicle animals, *P* < 0.05. Sex adjusted mean differences were: GluA1 0.15 (95% CI: 0.01–0.29; *P* = 0.036), GluA2 0.40 (95% CI: 0.29–0.50; *P* < 0.001), pKv2.1 0.26 (95% CI: 0.16–0.37; *P* < 0.001), p-CaMKII −0.27 (95% CI: −0.18 to −0.37; *P* < 0.001), and BDNF −0.17 (95% CI: −0.21 to −0.03; *P* = 0.017). Treatment (saline vs. 2R,6R-HNK by sex differences were GluA1, *P* = 0.683, GluA2, *P* = 0.204, p-Kv2.1, *P* *=* 0.001, p-CaMKII, *P* = 0.107, BDNF, *P* = 0.227. p-Kv2.1 effect greater in male than female mice. Differences not adjusted for multiple comparisons. Table: Representative immunoblots from the hippocampus of 3 mice treated with saline and 3 treated with (2R,6R)-HNK. Blots of protein of interest and GAPDH (housekeeping protein) are taken from the same lane on the chromatograph. (2R,6R)-HNK indicates (2R,6R) hydroxynorketamine; BDNF, brain-derived neurotrophic factor; CI, confidence interval; GAPDH, glyceraldehyde 3-phosphate dehydrogenase; GluA1, glutamate ionotropic receptor (AMPA) type subunit 1; GluA2, glutamate ionotropic receptor (AMPA) type subunit 2; p-CaMKII, phosphorylated-calcium/calmodulin-dependent protein kinase II; pKv2.1, phosphorylated voltage gated potassium channel 2.1.

**Figure 6:**
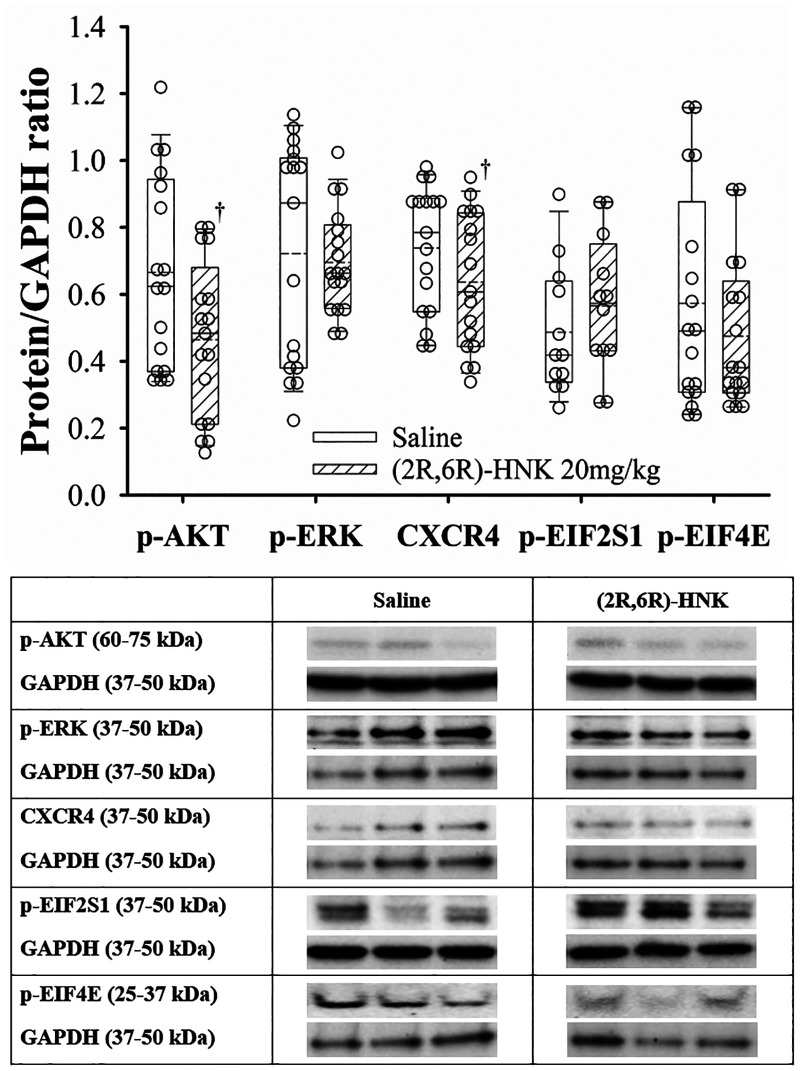
Dot and box plots of hippocampal protein/GAPDH ratios for saline and (2R,6R)-HNK treated animals following disk puncture surgery. The solid line is the median, the dashed line is the mean, the box represents the 25th to 75th percentiles, the whiskers represent the 10th and 90th percentiles, and the solid circle represents the 5th and 95th percentiles. † =(2R,6R)-HNK different from saline vehicle animals, *P* < 0.05. Weight and sex adjusted mean difference in CRCX4 −0.09 (95% CI: −0.03 to −0.17; *P* = 0.007), p-AKT −0.19 (95% CI: −0.09 to −0.29; *P* < 0.001), p-EIF2S1 0.09 (95% CI: −0.05 to 0.24; *P* = 0.214), p-EIF4E −0.08 (95% CI: −0.21 to 0.05; *P* = 0.229), and p-ERK(1/2) −0.02 (95% CI: −0.11 to 0.02; *P* = 0.665). Treatment (saline vs. 2R,6R-HNK by sex differences were CRCX4, *P* = 0.325, p-AKT, *P* = 0.405, p-EIFS2I, *P* *=* 0.283, p-EIF4E, *P* = 0.106, p-ERK(1/2), *P* *<* 0.001. p-ERK(1/2) effect greater in male than female mice. Differences not adjusted for multiple comparisons. Table: Representative immunoblots from the hippocampus of 3 mice treated with saline and 3 treated with (2R,6R)-HNK. Blots of protein of interest and GAPDH (housekeeping protein) are taken from the same lane on the chromatograph. (2R,6R)-HNK indicates (2R,6R) hydroxynorketamine; CRCX4, CXC chemokine receptor 4; CI, confidence interval; GAPDH, glyceraldehyde 3-phosphate dehydrogenase; p-AKT, phosphorylated protein kinase B; p-EIF2SI, phosphorylated eukaryotic translation initiation factor 2 subunit 1; p-EIF4E, phosphorylated eukaryotic translation initiation factor 4e; p-ERK(1/2), phosphorylated extracellular signal regulated kinase 1 and 2.

In the hippocampus, (2R,6R)-HNK treated mice demonstrated higher ratios for GluA1, GluA2, p-Kv2.1 and p-CaMKII to GAPDH proteins and a lower ratio for BDNF/GAPDH compared with saline treated mice (Figure 5). In addition, (2R,6R)-HNK treatment decreased the ratios of CXCR4 and p-AKT to GAPDH but did not affect the ratios of p-EIFS2I, p-EIF4E or p-ERK(1/2) to GAPPDH compared to saline treatment (Figure 6). Full blots available in Supplementary Figure S6.

### Effect of (2R,6R)-HNK administration on protein expression in the DRG

**Figure 7:**
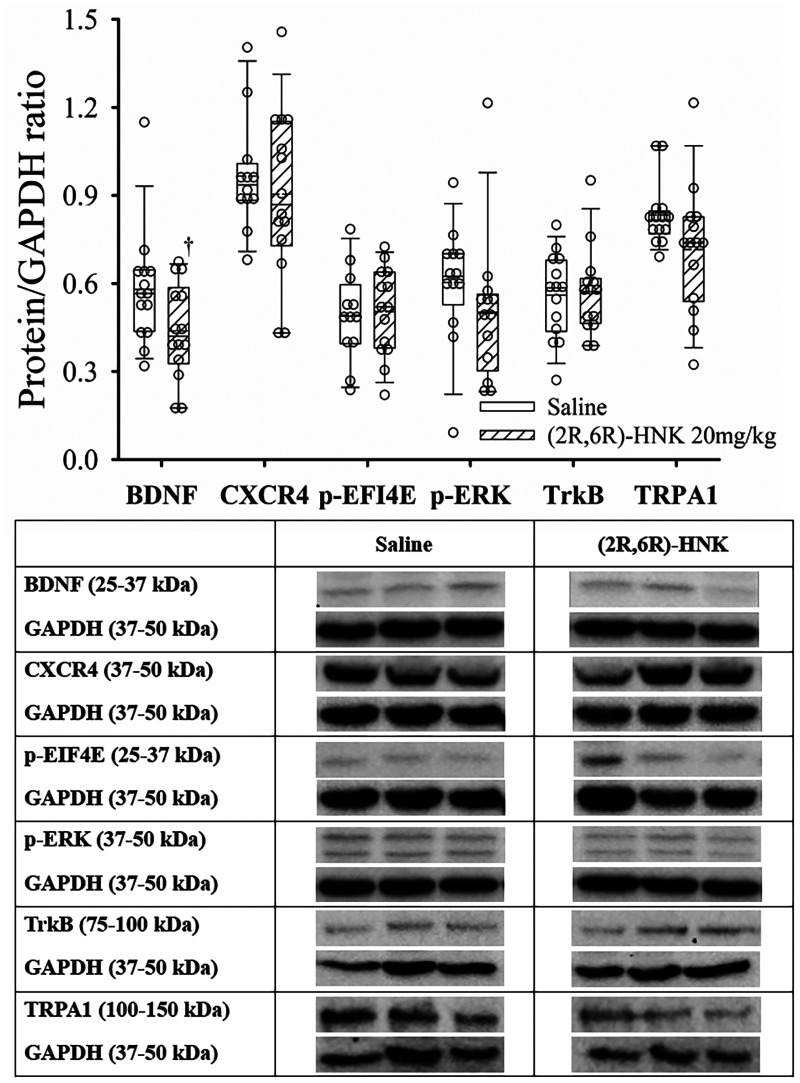
Dot and box plots of dorsal root ganglion protein/GAPDH ratios for saline and (2R,6R)-HNK mice following disk puncture surgery. The solid line is the median, the dashed line is the mean, the box represents the 25th to 75th percentiles, the whiskers represent the 10th and 90th percentiles, and the solid circle represents the 5th and 95th percentiles. † =(2R,6R)-HNK different from saline vehicle animals, *P* < 0.05. Sex adjusted mean difference were: BDNF difference −0.14 (95% CI: −0.26 to −0.47, *P* = 0.021), CRCX4 difference −0.04 (95% CI: −0.21 to 0.13, *P* = 0.613), p-EIF4e difference 0.02 (95% CI: −0.12 to 0.09, *P* = 0.745), p-ERK(1/2) difference −0.10 (95% CI: −0.26 to 0.06, *P* = 0.227), TrkB difference 0.01 (95% CI: −0.07 to 0.09, *P* = 0.817), TRPA1 difference −0.12 (95% CI: −0.23 to 0.00, *P* = 0.055). Treatment (saline vs. 2R,6R-HNK by sex differences were BDNF, *P* = 0.105, CRCX4, *P* = 0.778, p-EIF4E, *P* = 0.798, p-ERK(1/2), *P* *=* 0.906, TrkB, *P* = 0.461, TRPA1, *P* *=* 0.186. Table: Representative immunoblots from the dorsal root ganglion of 3 mice treated with saline and 3 treated with (2R,6R)-HNK. Blots of protein of interest and GAPDH (housekeeping protein) are taken from the same lane on the chromatograph. (2R,6R)-HNK indicates (2R,6R) hydroxynorketamine; BDNF, brain-derived neurotrophic factor; CI, confidence interval; GAPDH, glyceraldehyde 3-phosphate dehydrogenase; CRCX4, CXC chemokine receptor 4; p-EIF4E, phosphorylated eukaryotic translation initiation factor 4e; p-ERK(1/2), phosphorylated extracellular signal regulated kinase 1 and 2; TrkB, tyrosine protein kinase B; TRPA1, transient receptor potential ankyrin 1.

At the DRG, (2R,6R)-HNK treated mice demonstrated a lower ratio for BDNF/GAPDH, but did not significantly affect the ratios of CXCR4, EFI4E, p-ERK(1/2), TrkB or TRPA1 to GAPDH (Figure 7). Full blots available in Supplementary Figure S7.

## Discussion

The important new behavioral findings of this study are the dose dependence of the (2R,6R)-HNK antiallodynic response, with a non-significant difference in the EC_50_ anti-allodynic dose in male & female DP mice, and that co-administration of (2R,6R)-HNK with meloxicam increased antinociception of the combination compared with meloxicam alone. The antiallodynic effect of (2R,6R)-HNK is blocked by pretreatment with a brain penetrant AMPA agonist but not with naloxone (15, 16). Like our prior study (2R,6R)-HNK antiallodynic activity was associated with increased hippocampal protein ratios of GluA1, GluA2, p-Kv2.1 and p-CaMKII, with a reduced ratio of BDNF (15). Novel to this study was the reduced p-AKT/GAPDH ratio which was not seen in naïve, spared nerve injury, tibial fracture or hind paw incision mice (15). Our findings also demonstrated that (2R,6R)-HNK reduced the c-Fos upregulation at the spinal cord level, and we observed a reduction in the BDNF/GAPDH ratio at the dorsal root ganglion but did not detect differences in the CXCR4, TRPA1, TrkB, p-ERK, and p-EIF4E to GAPDH ratios.

The model used in this study is of chronic inflammatory pain induced by DP. Prior studies demonstrating analgesia with (2R,6R)-HNK have used pain-stimulated behavior models including hind paw incision, sciatic nerve injury, and bone fracture (14, 15, 16). Studies evaluating acute antinociceptive efficacy of (2R,6R)-HNK, such as thermal withdrawal latencies and noxious chemical injuries, have not reliably demonstrated antinociceptive activity, suggesting that the benefit of (2*R*,6*R*)-HNK in chronic pain states may be a result of producing long-term changes in the inflammatory response or neuroplasticity rather than on direct modulation of the nociceptive stimulus (22). The differences in the ratios of protein pathways at the dorsal root ganglion, spinal cord and in the hippocampus observed in our studies as well as changes in prelimbic cortex to periaqueductal gray connectivity modify central sensitization as a potential mechanism of alleviation of neuropathic pain (23).

We found that the antiallodynic effect of (2R,6R)-HNK was not blocked by the opioid receptor antagonist naloxone; however, recent studies suggest that the 6-hydroxynorketamine metabolites of ketamine act as positive allosteric modulators of the opioid receptors enhancing met-enkephalin actions on the mu opioid receptor which may represent an additional analgesic action of (2R,6R)-HNK (24). This effect could also explain the prolonged analgesic effect of ketamine and ketamine metabolites. The half-life of (2*R*,6*R*)-HNK in brain and plasma is less than 1 h and the drug is eliminated by the onset or during the sustained antinociceptive effects seen in our study (25).

The exact molecular mechanisms and key target sites by which (2R,6R)-HNK exerts its antinociceptive effect are unclear. In a novel murine model of chronic primary pain, Liu et al. demonstrated superior delayed antiallodynic effect of intrathecal (2R,6R)-HNK compared with intraperitoneal administration suggesting the central nervous system as the primary site of (2R,6R)-HNK activity (26). Mechanistically they found that (2R,6R)-HNK suppressed neuronal hypersensitivity by attenuating the upregulation of calcitonin gene related peptide (CGRP), transient receptor potential ankyrin 1 (TRPA1) or vanilloid-1 (TRPV1), and vesicular glutamate transporter-2 (VGLUT2) in peripheral nociceptive pathway. They also found that the c-Fos increase in multiple sites in the brain invoked by low-frequency percutaneous electrical nerve stimulation were blocked by pretreatment with (2R,6R)-HNK. Upregulation of spinal c-Fos has been demonstrated following sciatic nerve neuropathy and we observed upregulation of c-Fos following DP which was suppressed in the lumbar spinal cord with (2R,6R)-HNK treatment (27, 28). In contrast when administered intraperitoneally our findings suggest that (2R,6R)-HNK analgesic activity in DP mice did not produce a significant reduction in TRPA1 in the DGR.

(2R,6R)-HNK produces a weak antinociceptive effect initially (2–4 h) with a more substantial secondary effect at 24 h. The secondary effect can persist up to 72 h and we have found it to produce a more long-lasting effect with a series of 3 doses administered daily (19). To evaluate co-administration with another analgesic we need to find an analgesic that provided analgesia for 24 h. We were able to achieve an increase in PWT's with meloxicam at 24 h, but only with a high dose 10 mg/kg administered twice a day (29). We limited our study of the combination of meloxicam and (2R,6R)-HNK to only 3 days as longer-term administration of non-steroidal anti-inflammatory drugs in rodents can produce intestinal lesions and significant adverse effects (30). Nonetheless, our combination studies of meloxicam with (2R,6R)-HNK suggest that combination of (2R,6R)-HNK with a NSAID could have the potential to provide additive analgesia in clinical use.

A strength of our study is the large sample size and the demonstration of a dose dependent antiallodynic effect of (2R,6R)-HNK. Another strength is the evaluation of the combination of (2R,6R)-HNK with meloxicam demonstrating enhanced antinociception of the combination of (2R,6R)-HNK with a clinically relevant analgesic for low back pain. However, the findings of our study should only be interpreted within the context of its limitations. Preclinical pain models in mice may not accurately represent pain in humans, and effective treatments in mice may not translate to humans. We administered (2R,6R)-HNK via an IP route, so brain area–specific effects of the protein changes cannot conclusively suggest that the hippocampus is the specific site of the analgesic effects observed.

Despite many unanswered questions regarding the mechanism of (2R,6R)-HNK analgesia, clinical trials with this agent are underway. A Phase 1 study in healthy volunteers (NCT04711005) demonstrated (2R,6R)-HNK possessed a minimal adverse event profile and no serious adverse events at dose from 0.1 to 4 mg/kg (31). Cerebrospinal fluid examination confirmed (2R,6R)-HNK exposure within the central nervous system and quantitative electroencephalography demonstrated increased gamma power as a biomarker of clinical efficacy. Three clinical trials assessing (2R,6R)-HNK for neuropathic pain (NCT05864053), obsessive-compulsive disorder (NCT06575075) and treatment-resistant depression (NCT06511908) are listed on ClinicalTrials.gov, with the first of these currently open for recruitment.

## Conclusion

We found a dose dependent analgesic benefit of (2R,6R)-HNK and co-administration with meloxicam produces an enhanced anti-allodynic effect. There does not appear to be opioid receptor involvement in the anti-allodynic effect of (2R,6R)-HNK. Protein analysis suggests that (2R,6R)-HNK analgesic is associated with augmenting GluA1, GluA2, CaMKII, Kv2.1 and a reduction in BDNF protein ratios in hippocampus, decreased spinal cord c-Fos and reduced BNDF at the dorsal root ganglion. Clinical trials of this agent for treatment of pain and mental health disorders will be underway soon.

## Data Availability

The datasets presented in this study can be found in online repositories. The names of the repository/repositories and accession number(s) can be found below: https://data.mendeley.com/datasets/5dfxtxjk8f/1.
