# Supplementary material for: Behavioral and biochemical changes associated with the analgesic effects of (2R,6R)-hydroxynorketamine alone and in combination with meloxicam following disk puncture in mice
Source: Front Pain Res (Lausanne). 2025 Jun 12;6:1574474. doi: 10.3389/fpain.2025.1574474 (PMC12203739; doi:10.3389/fpain.2025.1574474)
Supplement: Supplementary file 1 [file Datasheet1.pdf]

Supplemental Table 1: List of drugs, reagents, supplies and equipment:

Description	*Catalog Number/ RRID*	*Supplier*
Disk Puncture Surgical Reagents & Tools		
Isoflurane	NDC66794-017-25	Piramal Enterprises Limited, Telangana, India.
f/air (Omnicon)	80120	Bickford Inc, Wales Center, NY, USA.
Surgical Tape	1527-2	3M, Saint Paul, MN, USA.
Povidone Iodine, USP 7.5% topical antiseptic solution)	NDC 67618-151-04	Purdue Pharma L.P., Stamford, CT
Bard-Parker™ Surgical Blades (#15)	371115	Aspen Surgical, Caledonia, MI, USA.
Cotton Tipped Applicators	MDS202055	Medline Inc, Mundelein, IL, USA.
25G hypodermic needle (BD PrecisionGlide Needle)	305122	Becton Dickinson and Company, Franklin, NJ, USA.
4-0 nylon suture (Ethilon)	1611G	Ethicon Inc., Norwalk, CA, USA.
Gentamicin sulfate (20mg/2mL) injection, USP	401898G	Fresenius Kabi, Lake Zurich, IL, USA.
Pharmacological Validation Studies		
Sodium Chloride 0.9% for injection 10mL vial	04888010	Hospira, Lake Forest, IL USA
(2R,6R)-Hydroxynorketamine	NCATs (Gift)	Maryland, USA
Naloxone Hydrochloride (4mg/10mL)	NDC67457-299-00	Mylan Pharmaceuticals Inc., VA, USA
AMPA antagonist (1,2,3,4-Tetrahydro-6-nitro-2,3-dioxobenzo [f]quinoxaline-7-sulfonamide (NBQX)	4798694	BioGems International, Inc., Westlake Village, CA, USA.
Meloxicam solution for injection	NDC46066-937-13	Pivetal, Loveland, CO USA 80538
Harvesting of Tissue and Sample Processing for Immunohistochemistry		
Paraformaldehyde (4% PFA)	441244-1KG	Sigma-Aldrich, Inc., St Louis, MO, USA.
10% Buffered Formalin Phosphate	SF100-4	Fisher Scientific, Chicago, IL, USA
Isopropyl Alcohol Solution 70%	8401	Decon Laboratories, Inc. King of Prussia, PA, USA.
Reagent Alcohol 90% (Histological Grade)	UN1987 (A962P-4)	Fisher Scientific, Chicago, IL USA.
95% Reagent Alcohol	HC13001GL	Fisher Scientific, Chicago, IL USA.
100% Reagent Alcohol	6900-1	StatLab, McKinney, TX, USA.
Xylenes reagent grade	X3P-1GAL	Fisher Scientific, Chicago, IL, USA.
Dioxane	D56S-4	Fisher Scientific, Chicago, IL, USA.
Paraffin (Surgipath, EM-400)	3801320	Leica, Chicago, IL USA

Microtome Blade (MX35 Premier)	3052835	Epredia, Kalamazoo, MI, USA.
Parapel	2300	Richard Allan Scientific, Kalamazoo, MI, USA.
Sodium Citrate	S-279	Fisher Scientific, Chicago, IL, USA
Phosphate buffer solution (10X solution)	BP3994	Fisher Scientific, Chicago, IL, USA
Triton X-100	T8787-250ML	Sigma-Aldrich, St. Louis, MO, USA
Normal Goat Serum	NS02L	EMD Millipore Corporation, Temecula, CA, USA.
DAPI (4',6-diamidino-2-phenylindole, dihydrochloride)	62247	Fisher Scientific, Chicago, IL, USA.
Fluoromount Mounting Medium	NB900- 66726	Novus, Novus Biologicals, LLC, Littleton, CO, USA.
Nikon Eclipse E600 Fluorescence Microscope	RRID: SCR_018606	Nikon, Tokyo, Japan
Camera, Jenopik Kapella 2.3 MP (Jenoptic ProgRes Capture Pro)	RRID: SCR_024489	Jenoptic, Jena, Germany.
ImageJ software	RRID:SCR_003070	Available at https://imagej.net/ij/index.html
Harvesting of Tissue and Sample Processing for Western Blotting		
Syn-Per reagent	87793	Thermo Fisher Scientific, Chicago, IL, USA.
N-PER Neuronal Protein Extraction reagent	87792	Thermo Fisher Scientific, Chicago, IL, USA.
Halt (Protease & Phosphatase Inhibitor Cocktail (100X)	78440	Thermo Fisher Scientific, Chicago, IL, USA.
Pierce (BCA Protein Assay Kit)	23227	Thermo Fisher Scientific, Chicago, IL, USA.
Spectramax M2e Microplate Reader	RRID: SCR_020297	Molecular Devices, San Jose, CA, USA.
RIPA buffer with Triton X-100 (1X)	J62725	Thermo Fisher Scientific, Chicago, IL, USA.
DL Dithiothreitol	D0632-1G	Sigma-Aldrich, Inc.; St Louis MO, USA
2x Laemmli Buffer	1610737	BIO-RAD, Hercules, CA
2-Mercaptoethanol	1610710	BIO-RAD, Hercules, CA
Mini-Protein TGX Gels (4-20%; 20µl/well)	4561095	BIO-RAD, Hercules, CA
Tris	1610716	BIO-RAD, Hercules, CA
30% Bis-Acrylamide	1610158;	BIO-RAD, Hercules, CA
SDS (Sodium Dodecyl Sulfate)	L3771-25G;	Sigma-Aldrich, Inc.; St Louis MO, USA
TEMED	BP150-20;	Fisher Scientific, Chicago, IL USA)
Ammonium Persulfate (APS)	1610700	BIO-RAD, Hercules, CA

10xTris/Glycine/SDS Buffer	1610772	BIO-RAD, Hercules, CA
Precision Plus Protein Kaleidoscope Standards	1610375	BIO-RAD, Hercules, CA
Trans-Blot Turbo RTA Transfer Kit, PVDF	1704273	BIO-RAD, Hercules, CA
BioRad Trans Blot Turbo system	SCR_023156	BIO-RAD, Hercules, CA
Methanol	A452SK-4	Fisher Scientific, Chicago, IL USA.
PVDF (Polyvinylidene fluoride) membrane	1620174	BIO-RAD, Hercules, CA
10xTris/Glycine Buffer	1610771	BIO-RAD, Hercules, CA
BSA (Bovine Serum Albumin)	BP1600-100	Fisher Scientific, Chicago, IL USA.
Immobilon Western (Chemiluminescent HRP Substrate)	WBKLS0500	Millipore, Burlington, MA, USA
Clarity Western ECL(Chemiluminescent) Substrate	170-5061	BIO-RAD, Hercules, CA USA.
Bio-Rad Gel 4000MP Versa Doc system	SCR_019678	BIO-RAD, Hercules, CA 94547
