# Supplementary material for: Behavioral and biochemical changes associated with the analgesic effects of (2R,6R)-hydroxynorketamine alone and in combination with meloxicam following disk puncture in mice
Source: Front Pain Res (Lausanne). 2025 Jun 12;6:1574474. doi: 10.3389/fpain.2025.1574474 (PMC12203739; doi:10.3389/fpain.2025.1574474)
Supplement: Supplementary file 2 [file Datasheet2.pdf]

Supplemental Table 2:

Antibodies for western blot and immunohistochemistry and mice RRIDs (Research Resource Identifiers) information used in this study:

Description	***Catalog number;Supplier***	***RRIDs***
C57BL/6J mice	Jackson Laboratory	RRID:MGI:5650797
Transient receptor potential ankyrin 1 (TRPA1)	PIPA146159; Thermo Fisher	RRID:AB_2209939
Protein C-Fos	ab208942; Abcam	RRID:AB_2747772
Neuronal nuclei marker (NeuN)	MAB377; Millipore<br>ABN 78; Millipore	RRID:AB_2298772<br>RRID:AB_10807945
Alexa Fluor 546	A11035; Invitrogen	RRID:AB_2534093
Alexa Fluor 555	A21427; Invitrogen	RRID:AB_2535848
Alexa Fluor 488	A11029; Invitrogen<br>A11008; Invitrogen	RRID:AB_2534088<br>RRID:AB_143165
Glutamate receptor subunit A1 (GluA1)	13185; Cell Signaling	RRID:AB_2732897
Glutamate receptor subunit A2 (GluA2)	13607: Cell Signaling	RRID:AB_2650557
Brain derived neurotrophic factor (BDNF)	47808; Cell Signaling	RRID:AB_2894709
Phosphorylated Ca2+/calmodulin-dependent protein kinase II (p-CaMKII)	12716S, Cell Signaling	RRID:AB_2713889
Phosphorylated voltage-activated potassium channel 2.1 (p-Kv2.1)	AB61107, Abcam	RRID:AB_2131640
Glyceraldehyde-3-phosphate dehydrogenase (GAPDH)	2118S; Cell signaling	RRID:AB_561053
Phosphorylated protein kinase B (p-AKT)	4060S, Cell Signaling	RRID:AB_2315049
Phosphorylated extracellular signal regulated kinase 1 and 2 (p-ERK(1/2))	9101S, cell Signaling	RRID:AB_331646
C-X-C motif chemokine receptor 4 (CXCR4)	ab181020, Abcam	RRID:AB_2910168
Phosphorylated eukaryotic translation initiation factor 2 subunit 1 (p-EIF2SI)	ab32157, Abcam	RRID:AB_732117
Phosphorylated eukaryotic translation initiation factor 4e (p-EIF4E)	ab76256, Abcam	RRID:AB_1523534
Tyrosine protein kinase B (TrkB)	AB187041, Abcam	RRID:AB_2892613
Immunoglobulin G (IgG)	7074S, Cell Signaling	RRID:AB_2099233

	7076S, Cell Signaling	RRID: AB_330924

Jackson Laboratories, Bar Harbor, ME, USA, Thermo Fisher Scientific, Waltham MA USA , Abcam, Waltham, MA, Cell Signaling Danvers, MA USA
