# Supplementary material for: Behavioral and biochemical changes associated with the analgesic effects of (2R,6R)-hydroxynorketamine alone and in combination with meloxicam following disk puncture in mice
Source: Front Pain Res (Lausanne). 2025 Jun 12;6:1574474. doi: 10.3389/fpain.2025.1574474 (PMC12203739; doi:10.3389/fpain.2025.1574474)
Supplement: Supplementary file 3 [file Datasheet3.pdf]

Supplemental text 1: Preparation and staining of paraffin sections for immunohistochemistry analysis.

Fixation & Dehydration of Murine Tissue for Embedding

On day 7, isolated spinal cord samples (L3-L5) were rinsed in deionized water and subsequently soaked in increasing percentages of ethyl alcohol (70%-100%). Dehydrated samples were then soaked in xylene for 30 minutes. The clearing process consisted of soaking samples in dioxane overnight, followed by xylene and xylene/paraffin. Paraffin embedded samples were cut at 7-10 μm thickness using a microtome and transferred into a warm water bath (40 °C). Spinal cord samples were collected onto slides and placed on a slide warmer until dry.

Paraffin Sections for Immunofluorescence

Slides were deparaffinized in two changes of xylene and hydrated in decreasing percentages of ethanol (70%-100%) and washed with deionized water. Samples were heated for 4 minutes in a sodium citrate buffer for the antigen retrieval process and allowed to cool for 1 hour before rinsing in 1X phosphate buffer solution (PBS) and permeabilized in 0.5% Triton X-100 in 1 x PBS. Nonspecific binding was blocked by incubating with 10% normal goat serum (NGS) for 1 hour at room temperature.
