# Supplementary material for: Behavioral and biochemical changes associated with the analgesic effects of (2R,6R)-hydroxynorketamine alone and in combination with meloxicam following disk puncture in mice
Source: Front Pain Res (Lausanne). 2025 Jun 12;6:1574474. doi: 10.3389/fpain.2025.1574474 (PMC12203739; doi:10.3389/fpain.2025.1574474)
Supplement: Supplementary file 4 [file Datasheet4.pdf]

Supplemental text 2: Protein and gel preparation for western blot analysis.

Protein was denatured with a 1:1 ratio of DL Dithiothreitol and 2x Laemmli buffer by heating which varied based on the size of protein targets of interest and tissue. For protein targets greater than 75 kDa, samples were denatured for 10 min at 115°C. For protein targets less than 75 kDa, samples were denatured for 8 min at 100°C.

The separating gel mixture for two gel plates was prepared using 3.025 mL ddH2O, 1.875 mL 1.5M Tris, pH 8.8, 2.475 mL 30% Bis-Acrylamide, 75 µL 10% SDS, 3.75 µL TEMED, and 37.5 µL 10% Ammonium Persulfate (APS), with TEMED and APS being added last and mixed thoroughly prior to dispensing into the prepared plates. The loading gel was prepared using 3.05 mL ddH2O, 1.25 mL 0.8M Tris pH 6.8, 650 µL 30% Bis-Acrylamide, 50 µL 10% SDS, 5 µL TEMED, and 25 µL 10% Ammonium Persulfate (APS), with TEMED and APS being added last and mixed thoroughly prior to dispensing into the prepared plates.

Glass plates (0.75 mm) were placed on a gel prep rig with the bottom of the plates sealed with tape. The separating gel mixture was dispensed into the gel plates using an electric pipette and 10 mL serological pipette. Ethanol (100%) was added to the top of the separating gel to ensure level gel formation and allowed to polymerize for approximately 30 minutes. After decanting the ethanol, the loading gel mixture was pipetted on to the gel plates on top of the polymerized separating gel. 15 well combs were placed in the loading gel mixture immediately after dispensing into prepared plates and gels were allowed to polymerize for approximately 20 minutes. Gels were stored in damp conditions at 4°C prior to use.
