# Supplementary material for: Behavioral and biochemical changes associated with the analgesic effects of (2R,6R)-hydroxynorketamine alone and in combination with meloxicam following disk puncture in mice
Source: Front Pain Res (Lausanne). 2025 Jun 12;6:1574474. doi: 10.3389/fpain.2025.1574474 (PMC12203739; doi:10.3389/fpain.2025.1574474)

Supplemental file
Full WB images Hippocampus

Behavioral and Biochemical Changes Associated with the Analgesic Effects of (2R,6R)-Hydroxynorketamine Alone and in Combination with Meloxicam Following Disk Puncture in Mice

Hippocampus: GluA1

Female

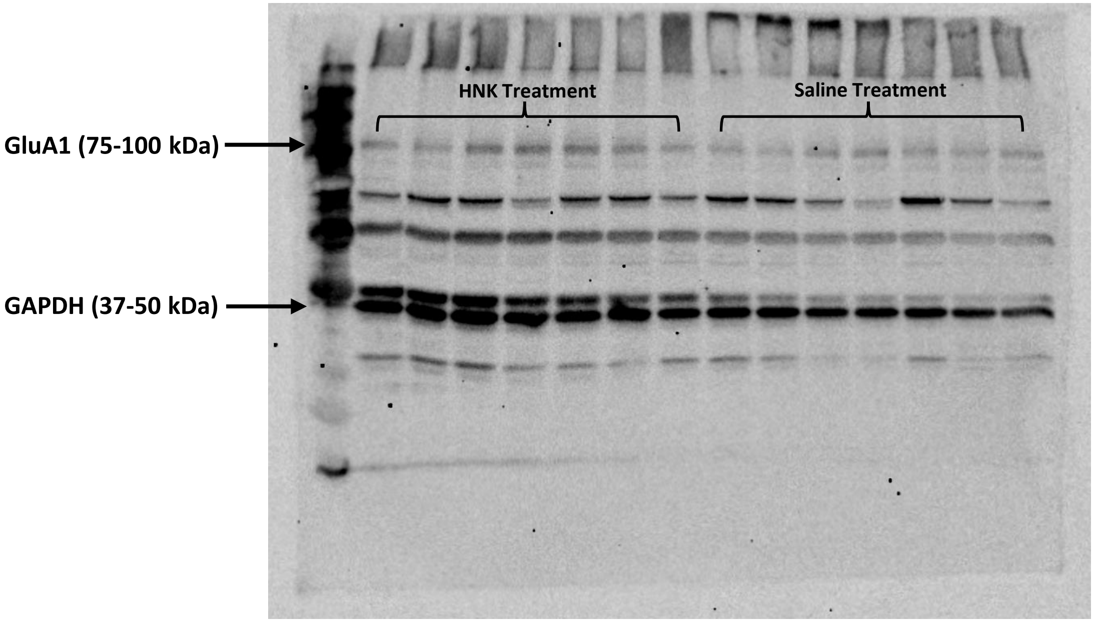

Male

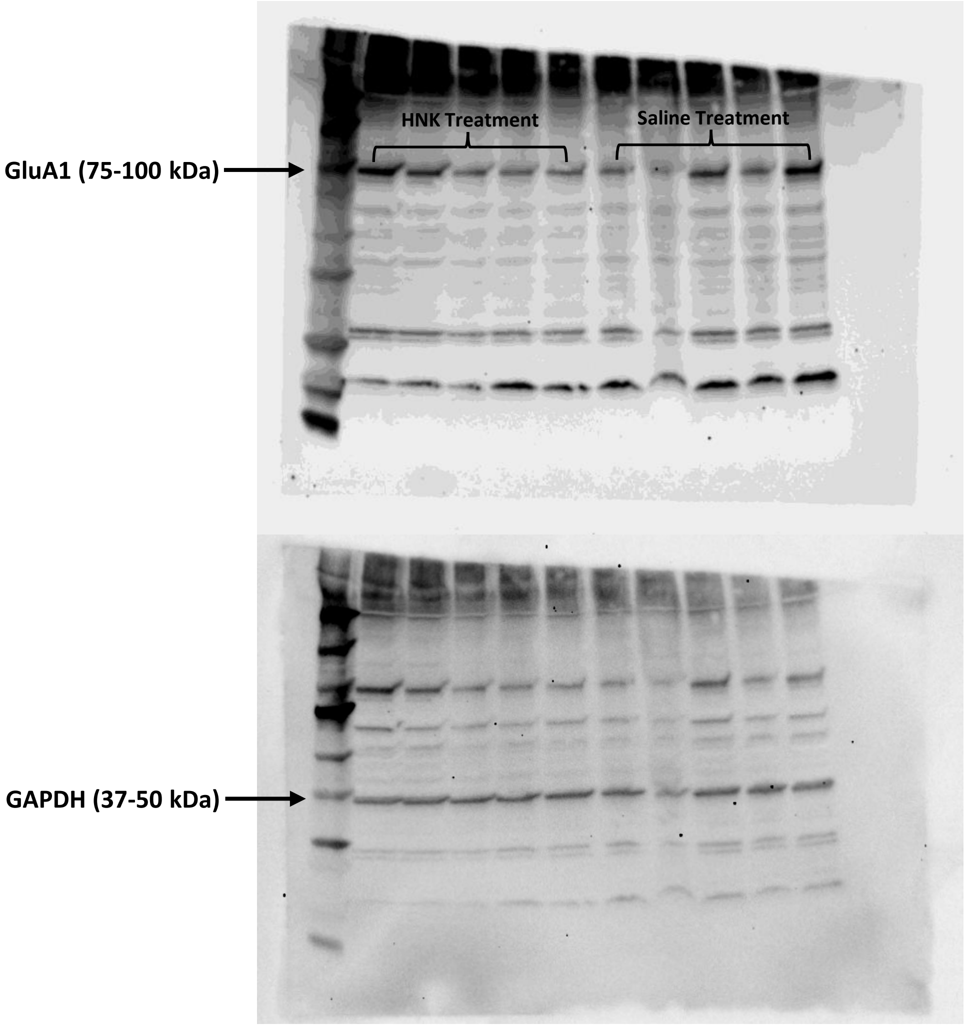

Hippocampus: GluA2

Female

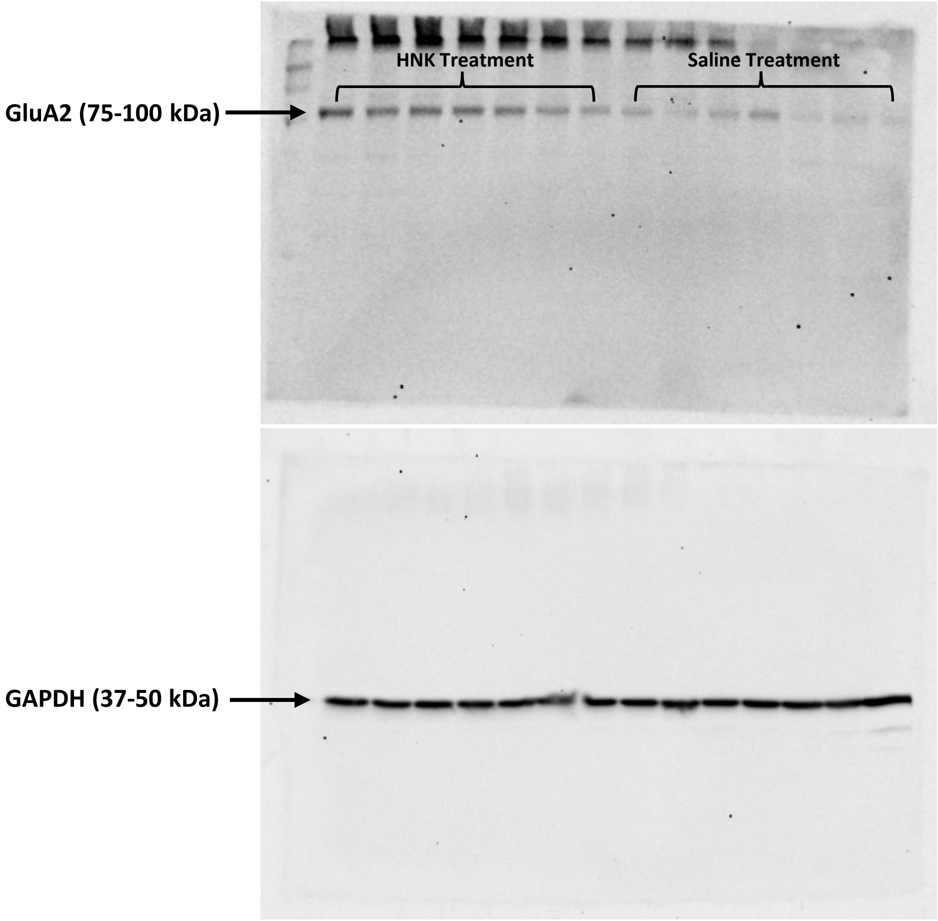

Male

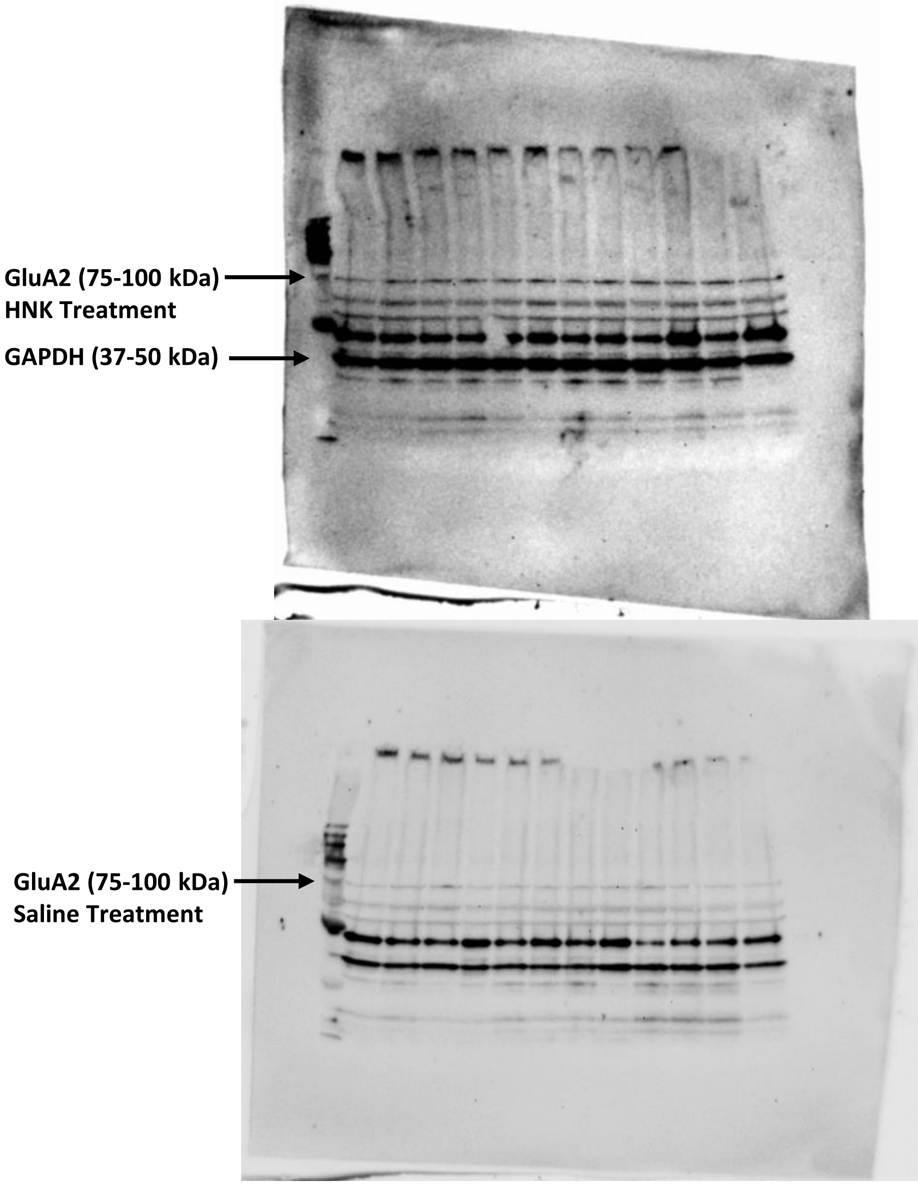

Hippocampus: p-Kv2.1

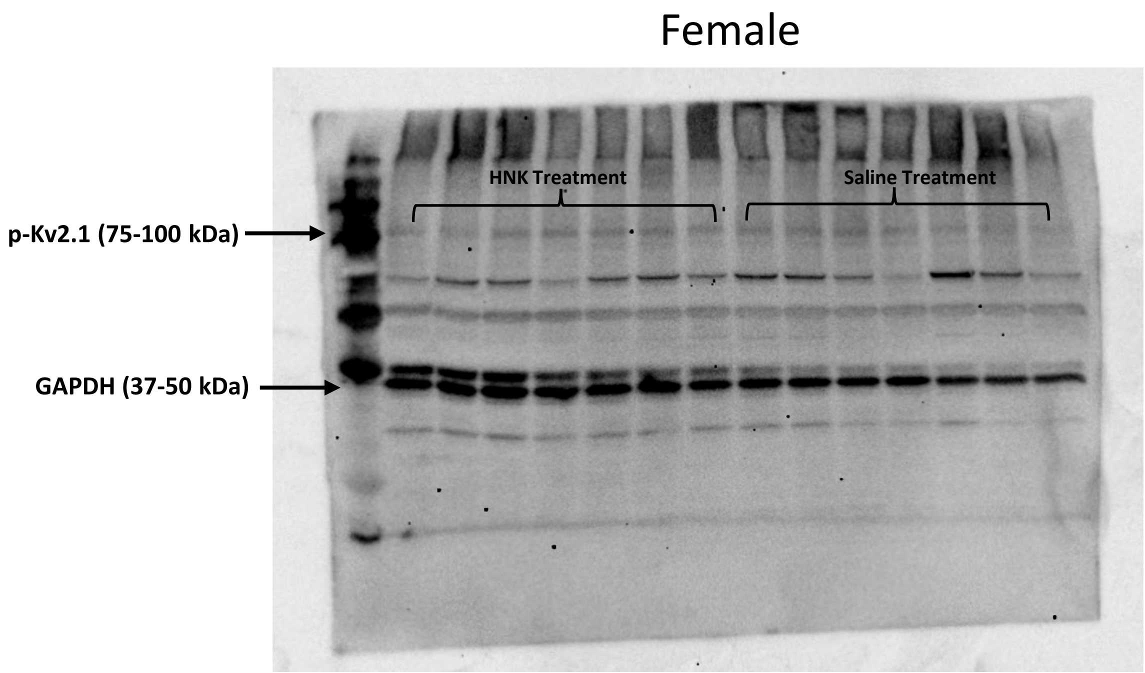

Hippocampus: p-Kv2.1

Male

p-Kv2.1 (75-100 kDa)
HNK Treatment

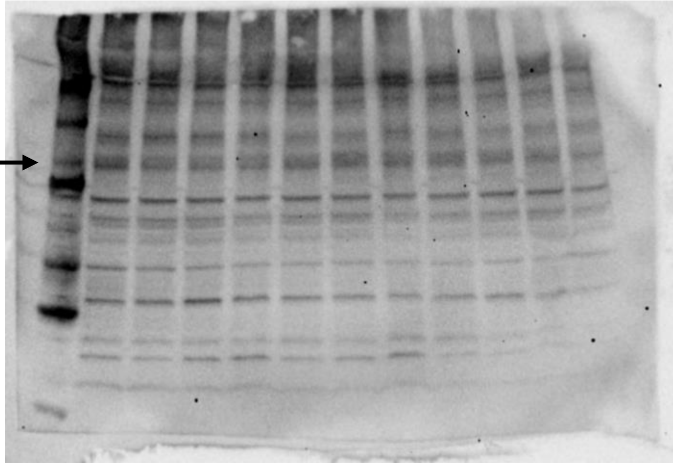

GAPDH (37-50 kDa)
HNK Treatment

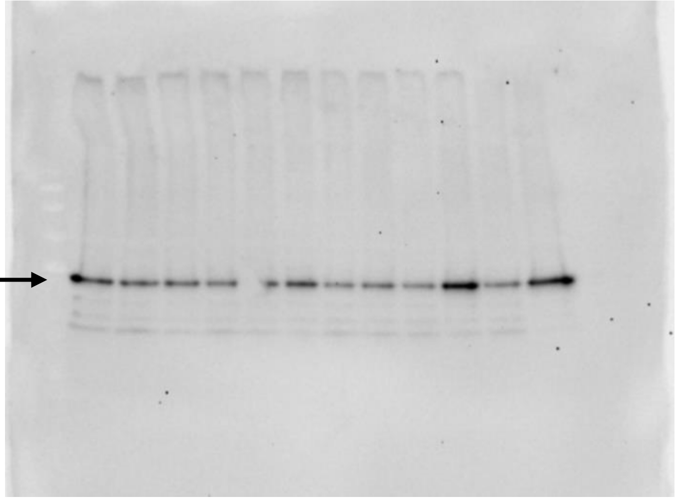

p-Kv2.1 (75-100 kDa)
Saline Treatment

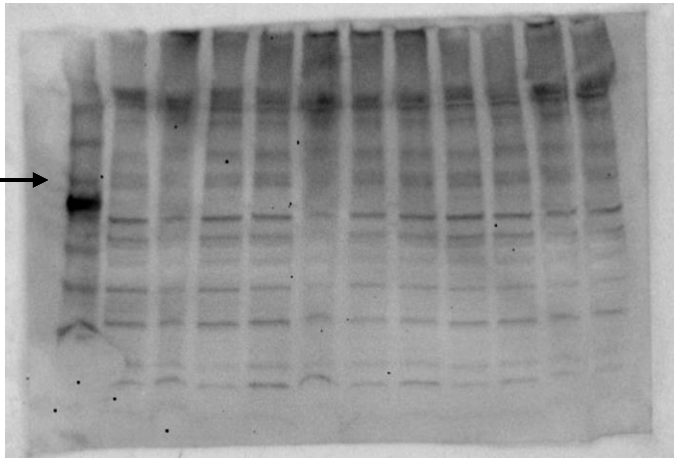

GAPDH (37-50 kDa)
Saline Treatment

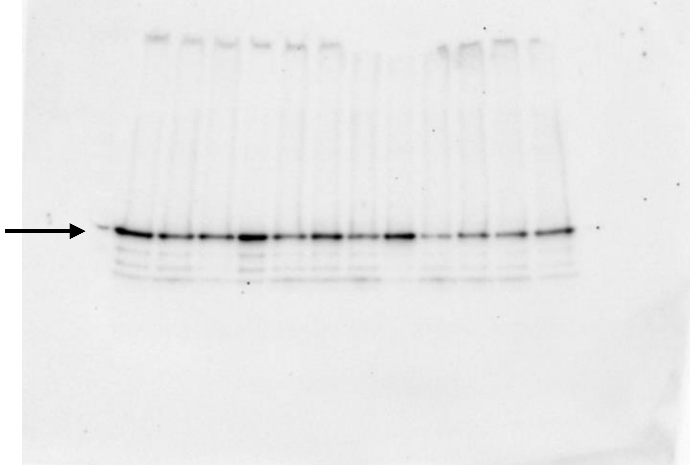

Hippocampus: CaMKII

Female

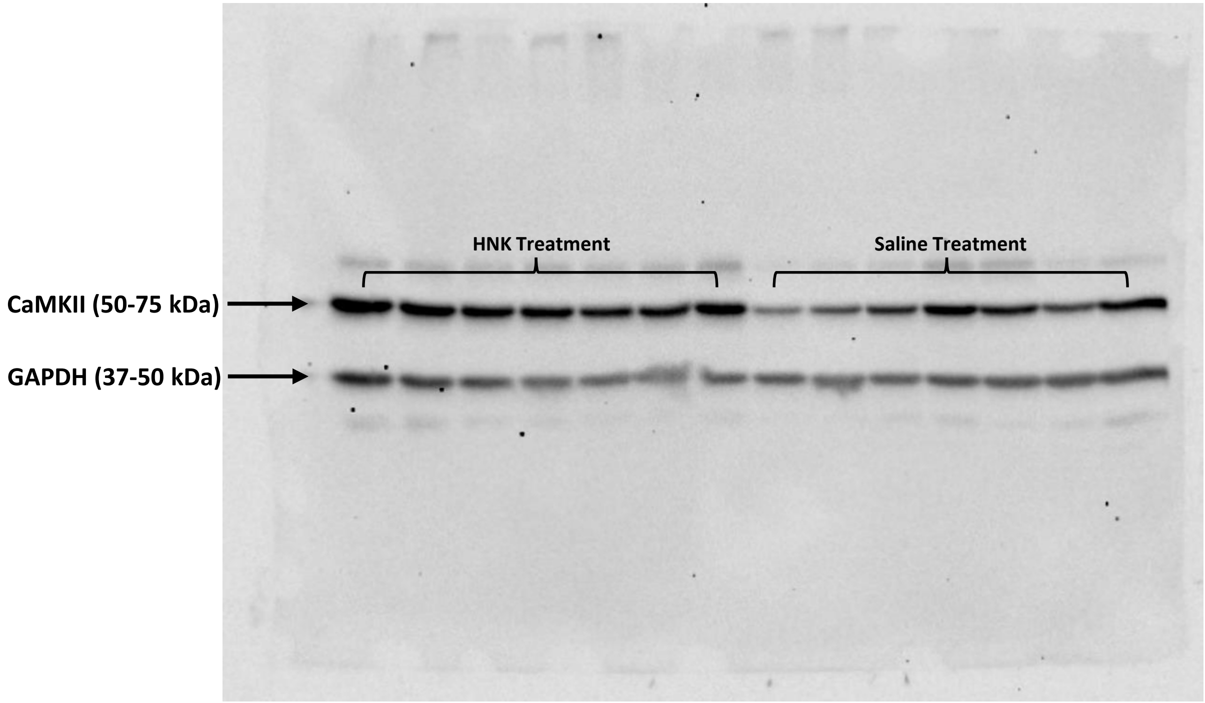

Male

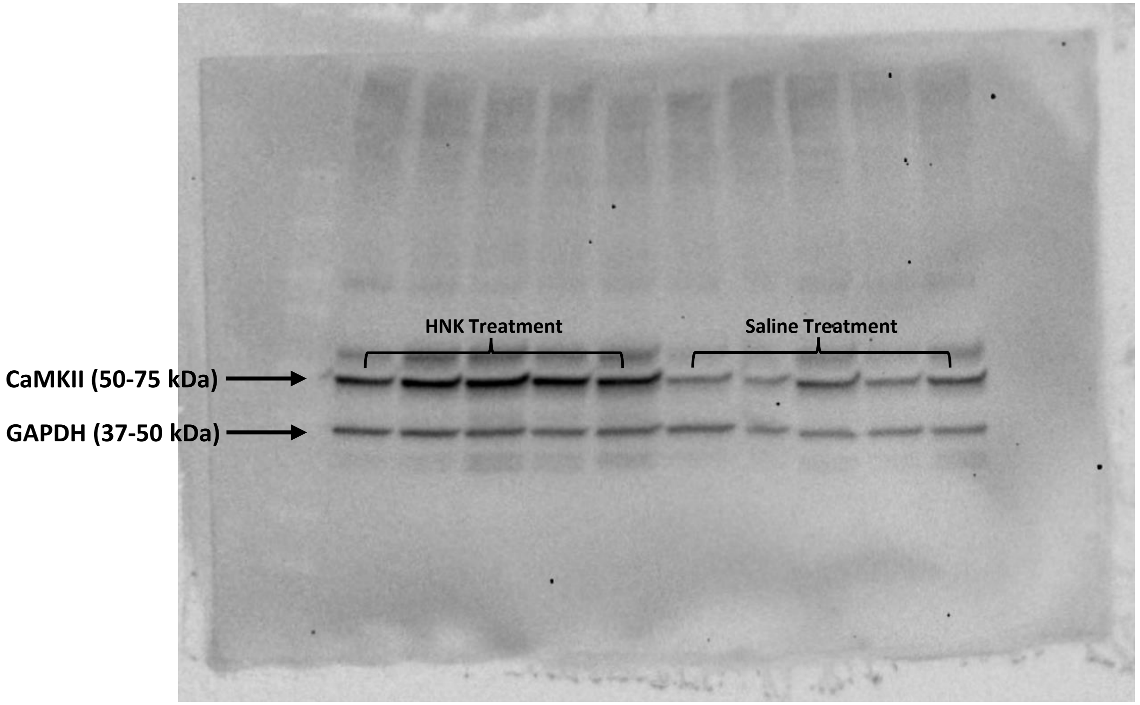

Hippocampus: BDNF

Female

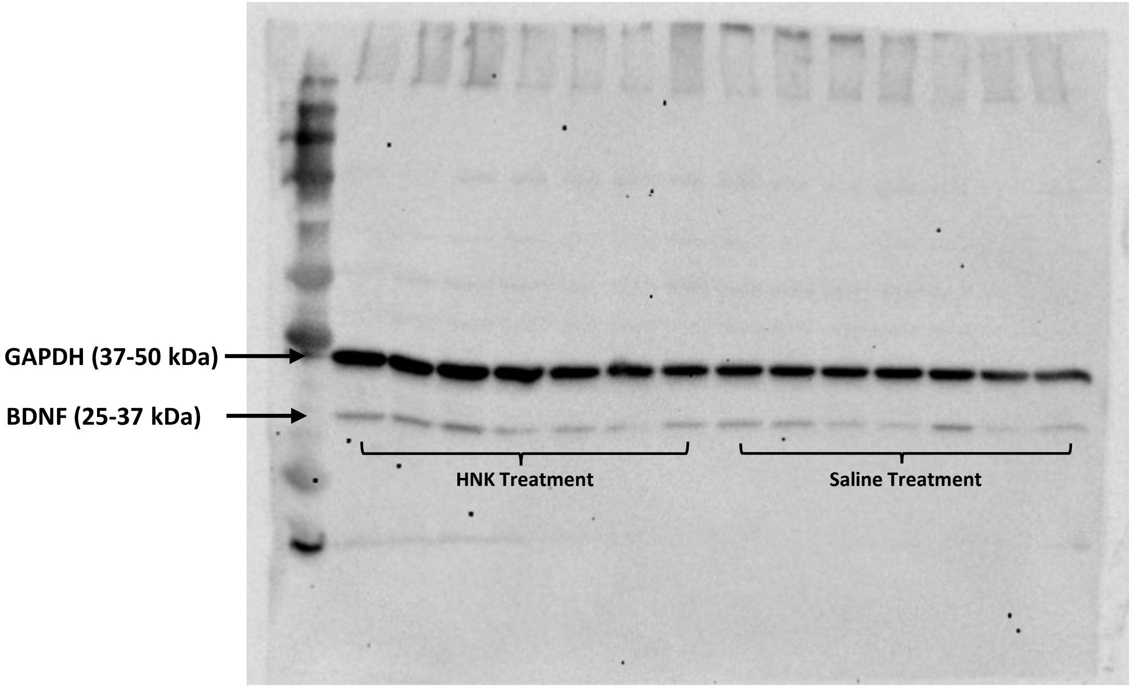

Hippocampus: BDNF

Male

BDNF (25-37 kDa)
HNK Treatment

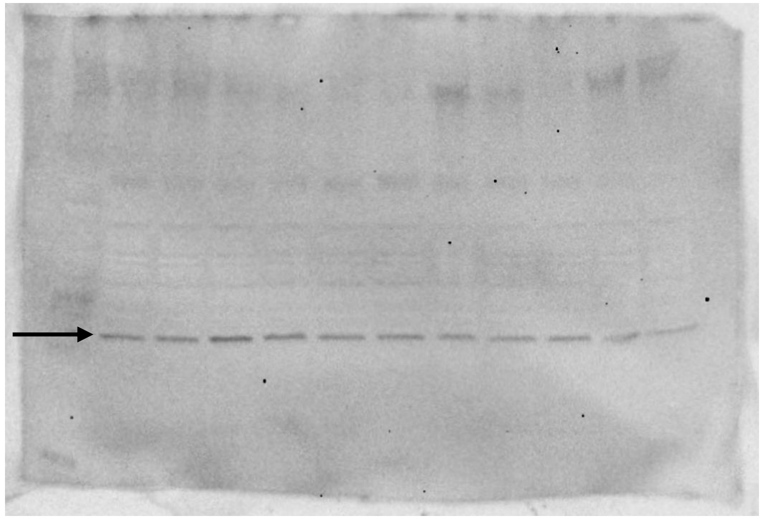

GAPDH (37-50 kDa)
HNK Treatment

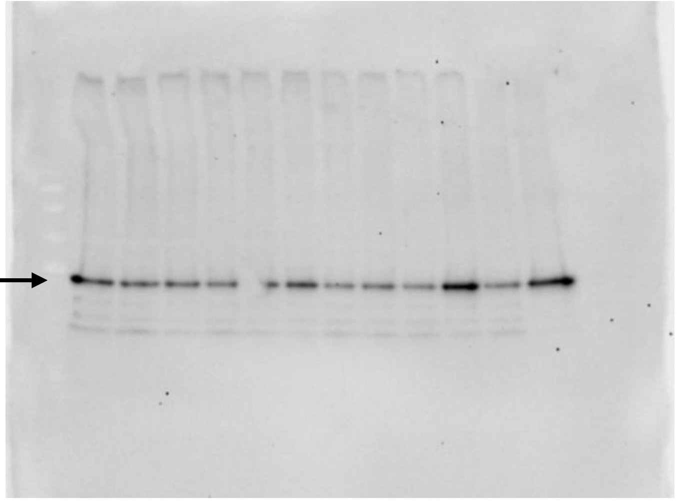

BDNF (25-37 kDa)
Saline Treatment

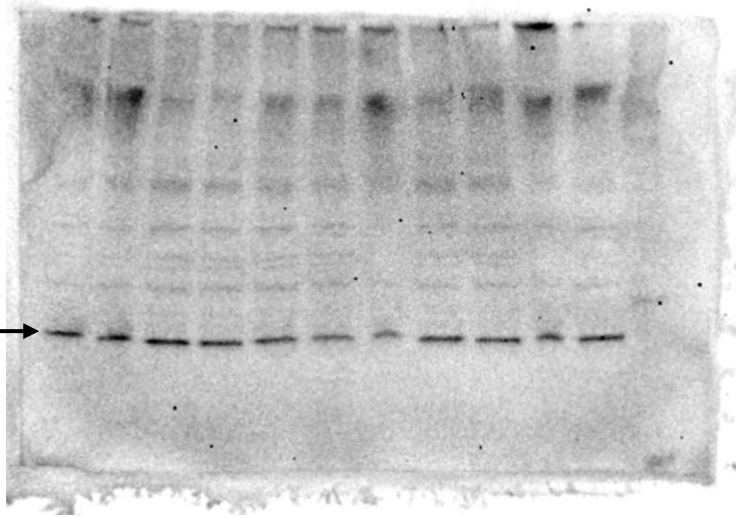

GAPDH (37-50 kDa)
Saline Treatment

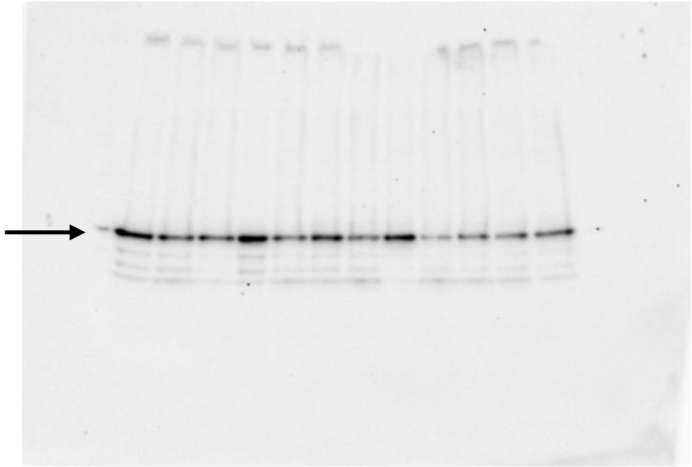

Supplementary

Hippocampus: AKT

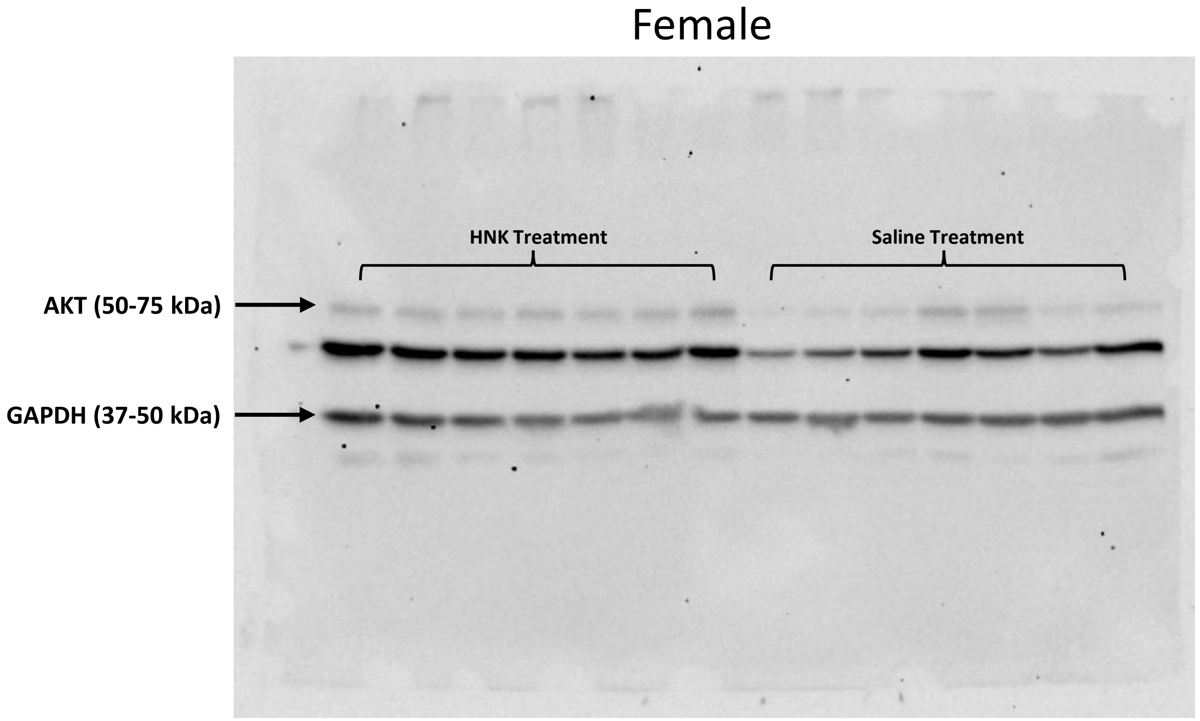

Supplementary

Hippocampus: AKT

Male

AKT (50-75 kDa)
HNK Treatment

GAPDH (37-50 kDa)
HNK Treatment

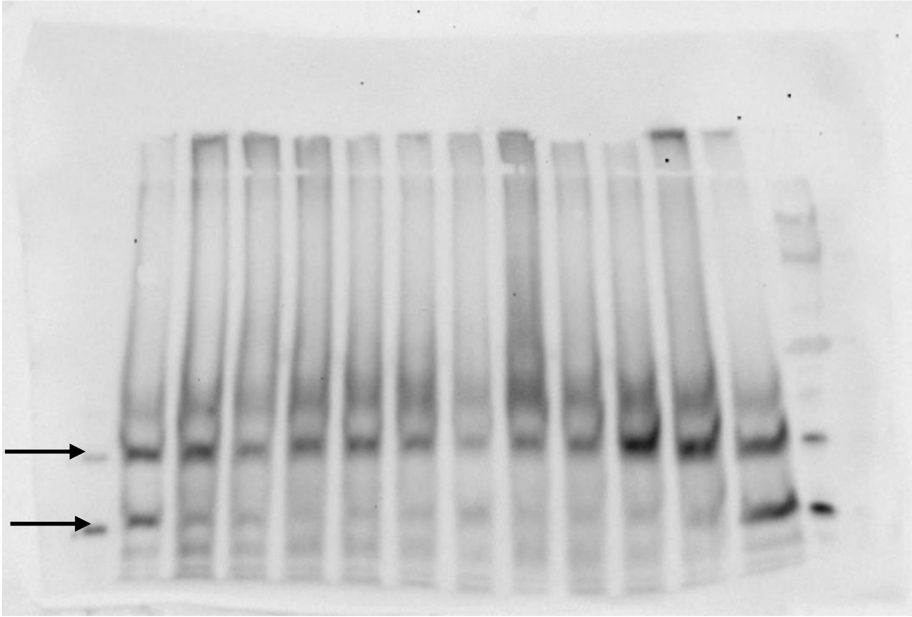

AKT (50-75 kDa)
Saline Treatment

GAPDH (37-50 kDa)
Saline Treatment

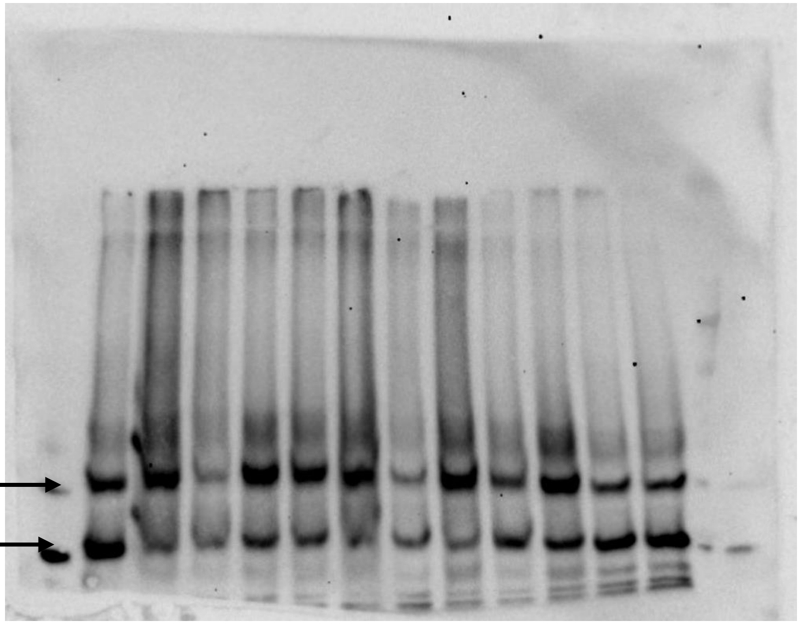

Supplementary

Hippocampus: CXCR4

Female

CXCR4 (37-50 kDa)

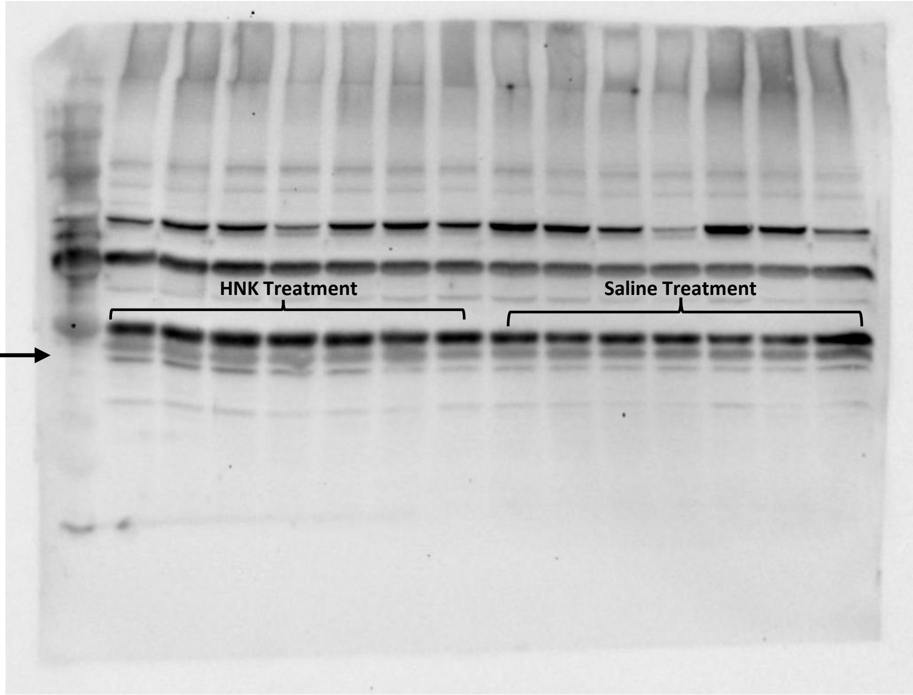

GAPDH (37-50 kDa)

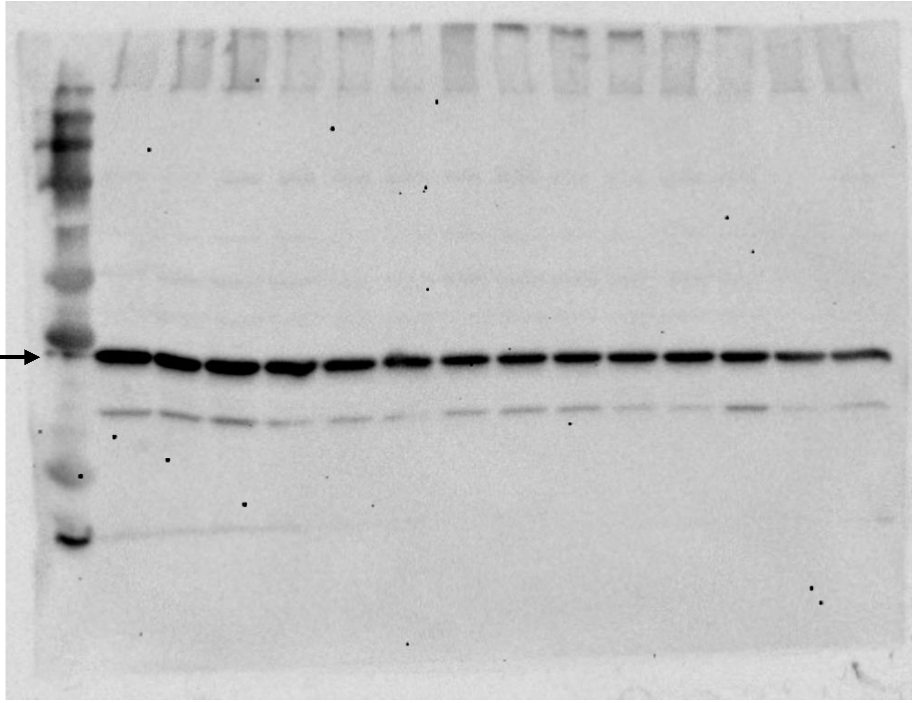

Supplementary

Hippocampus: CXCR4

Male

CXCR4 (37-50 kDa)
HNK Treatment

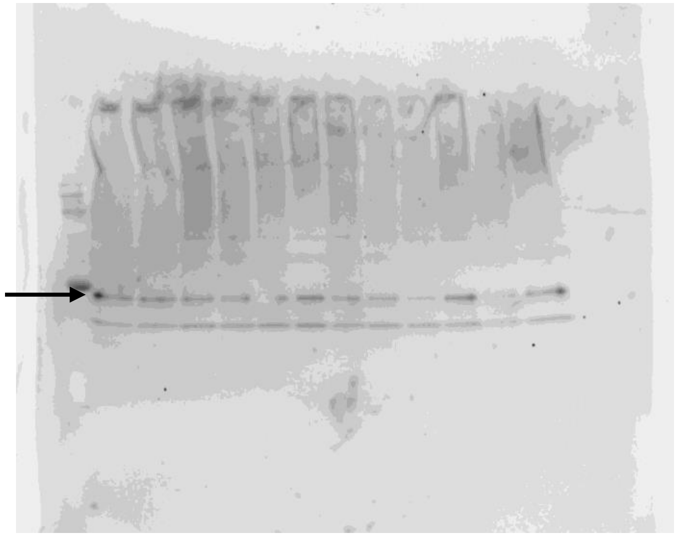

GAPDH (37-50 kDa)
HNK Treatment

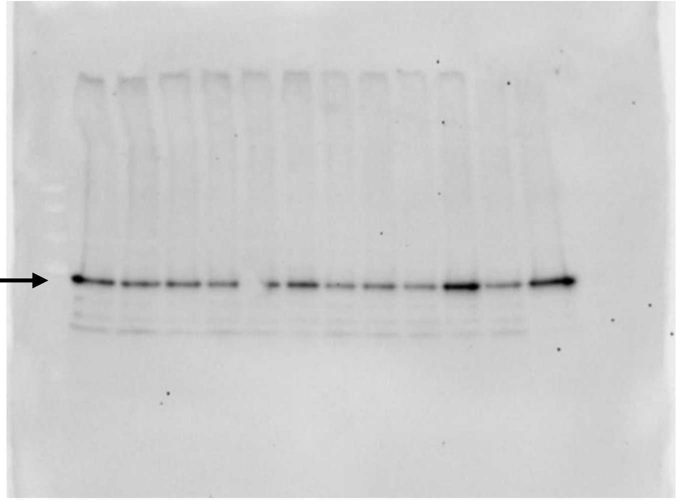

CXCR4 (37-50 kDa)
Saline Treatment

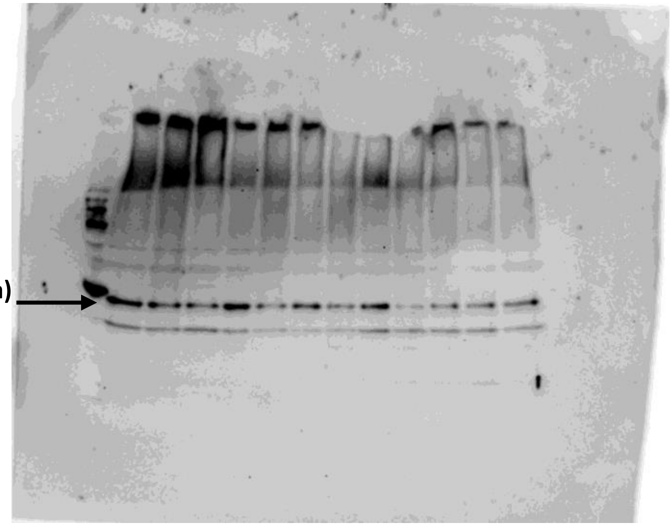

GAPDH (37-50 kDa)
Saline Treatment

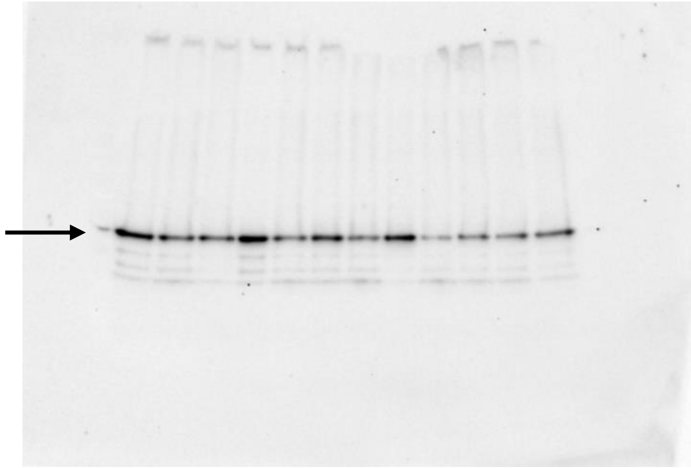

Supplementary

Hippocampus: p-ERK

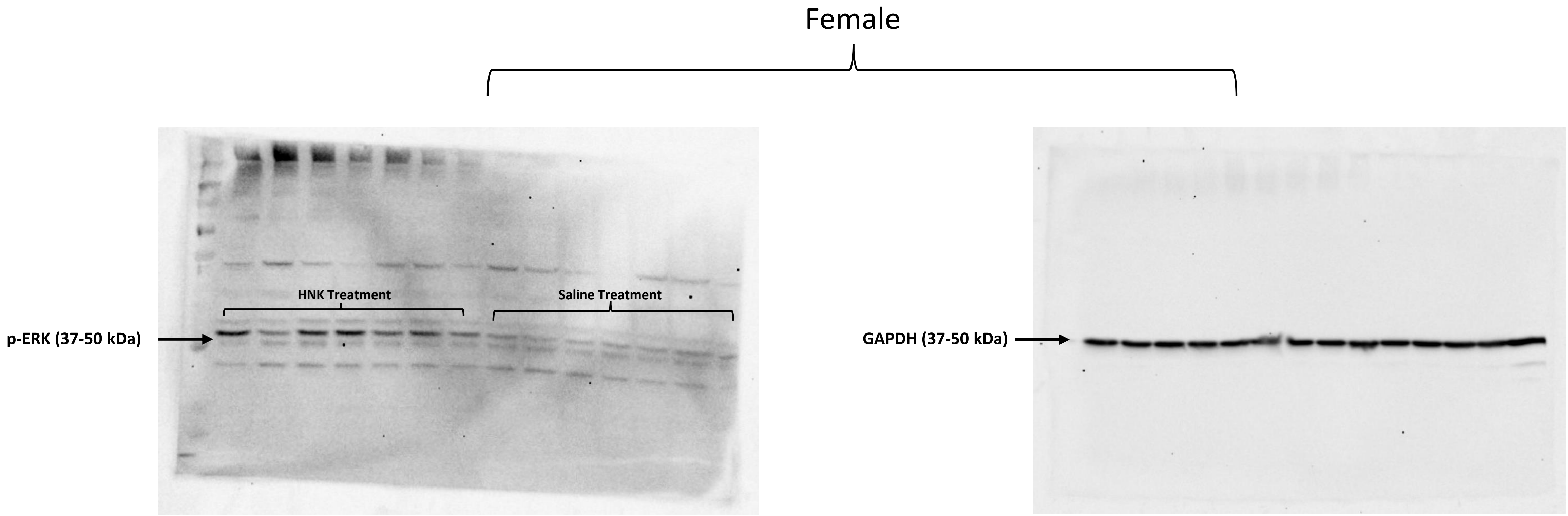

Supplementary

Hippocampus: p-ERK

Male

p-ERK (37-50 kDa)
HNK Treatment

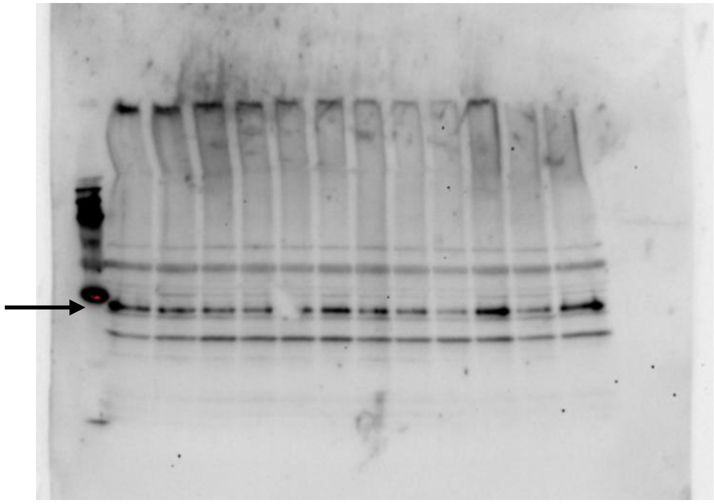

GAPDH (37-50 kDa)
HNK Treatment

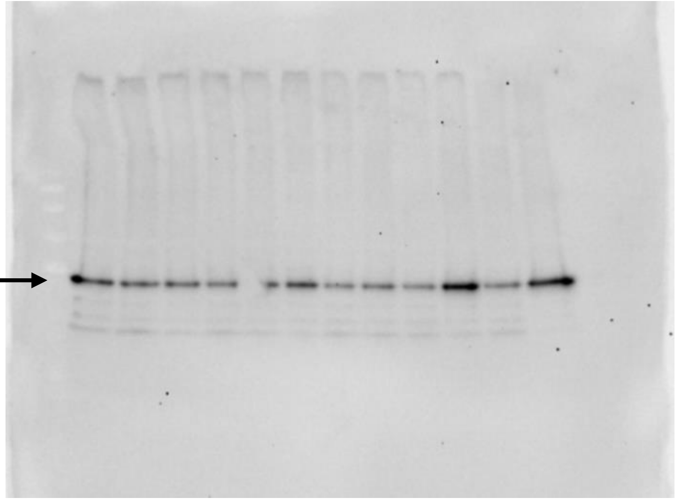

p-ERK (37-50 kDa)
Saline Treatment

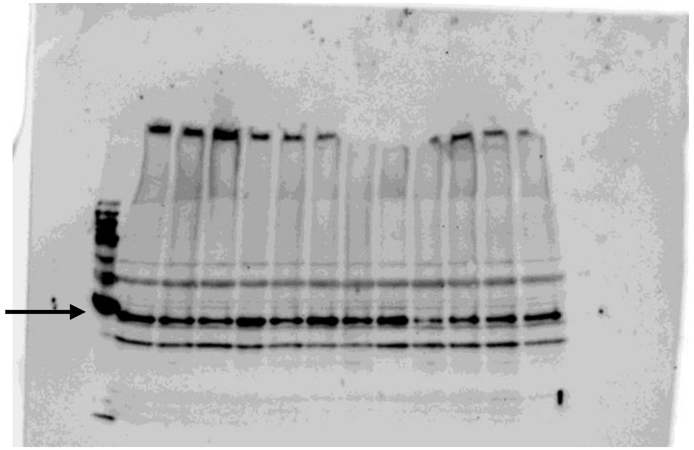

GAPDH (37-50 kDa)
Saline Treatment

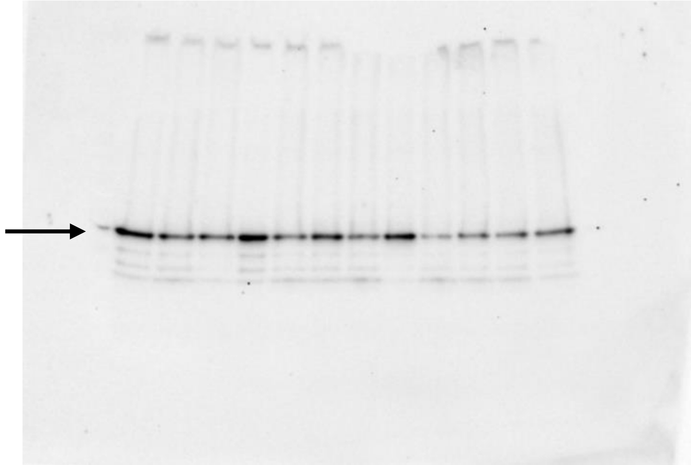

Supplementary

Hippocampus: p-EIF2S1

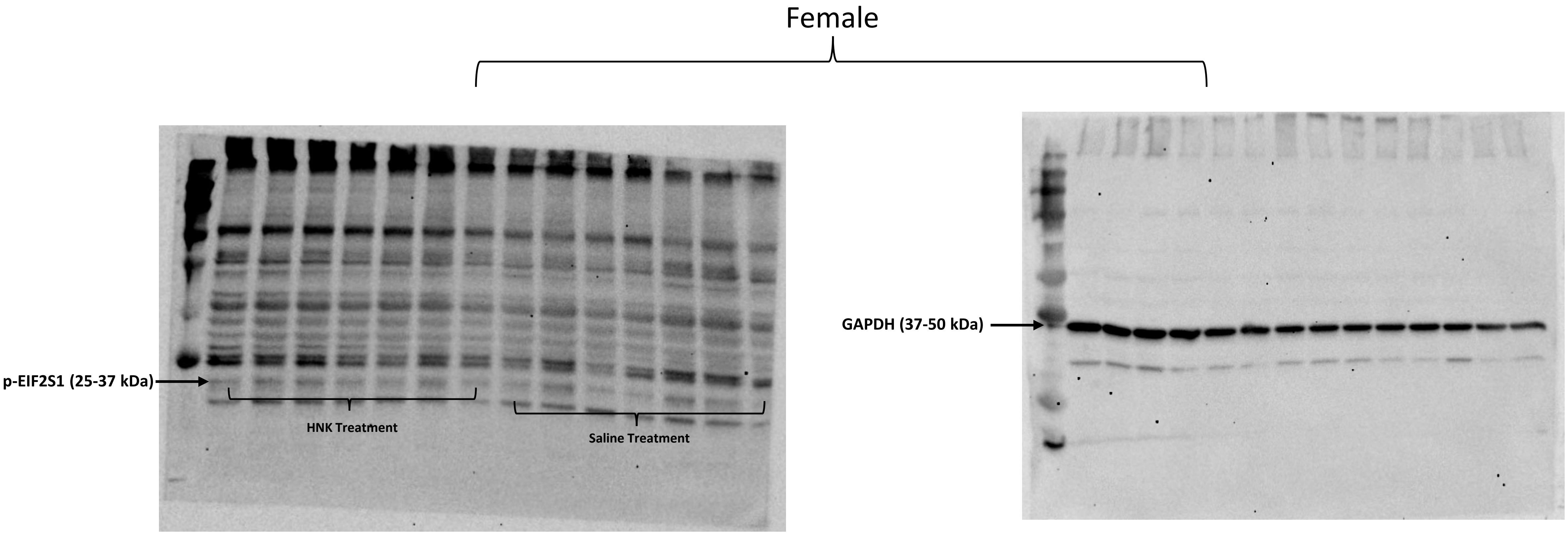

Supplementary

Hippocampus: p-EIF2S1

Male

p-EIF2S1 (25-37 kDa)

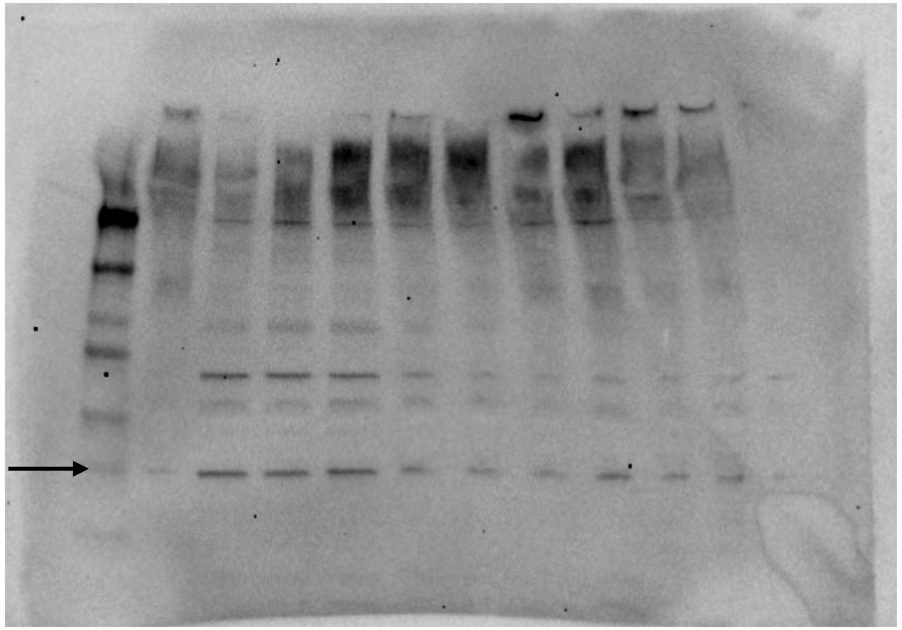

GAPDH (37-50 kDa)

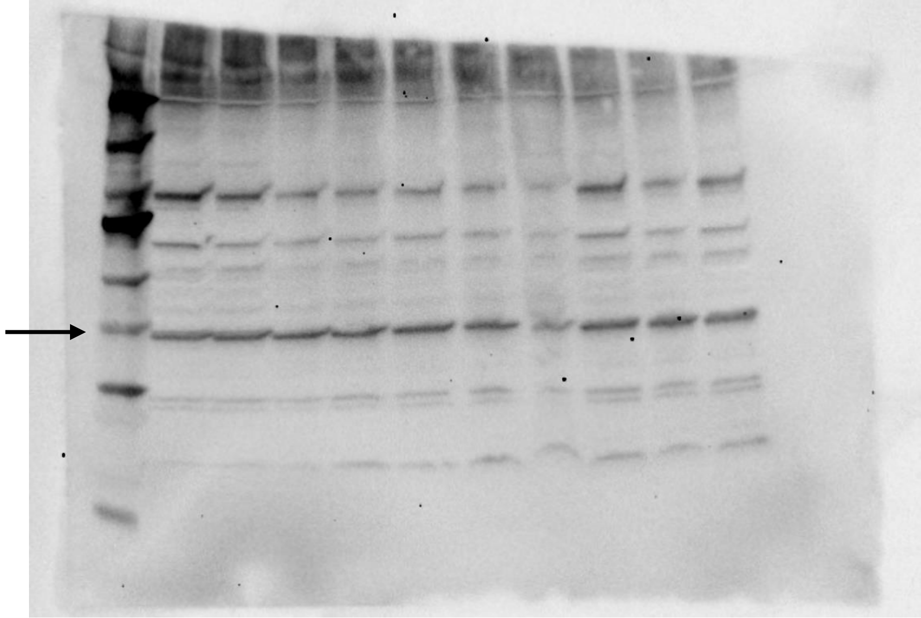

Supplementary

Hippocampus: p-EIF4E

Female

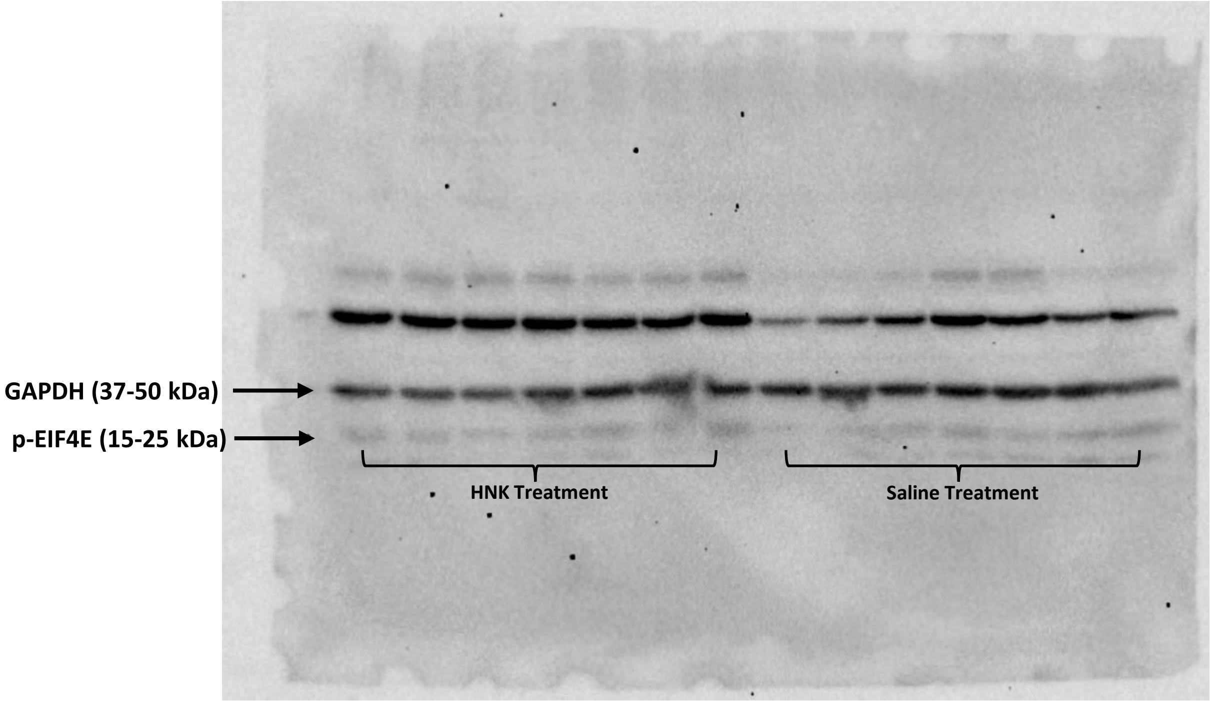

Supplementary

Hippocampus: p-EIF4E

Male

p-EIF4E (15-25 kDa)
HNK Treatment

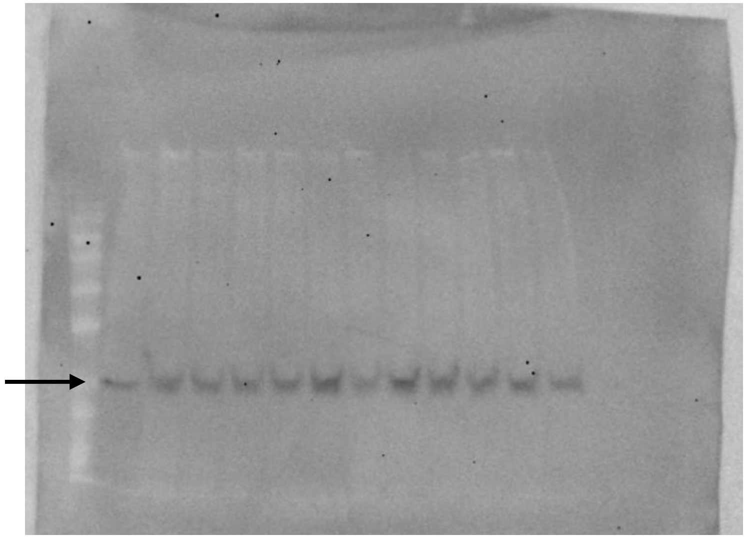

GAPDH (37-50 kDa)
HNK Treatment

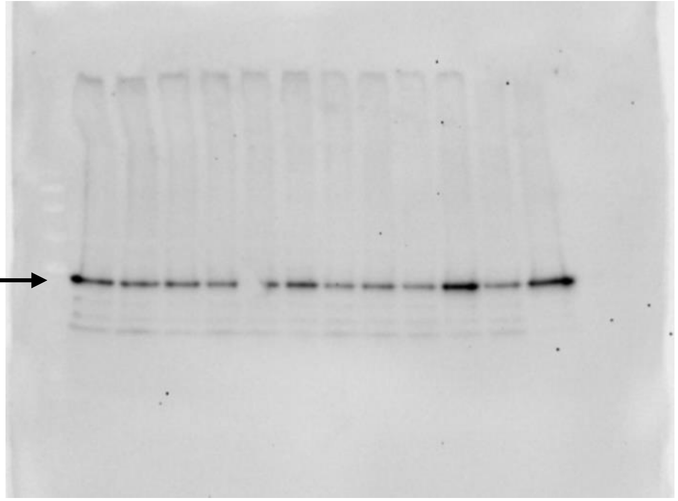

p-ERK (15-25 kDa)
Saline Treatment

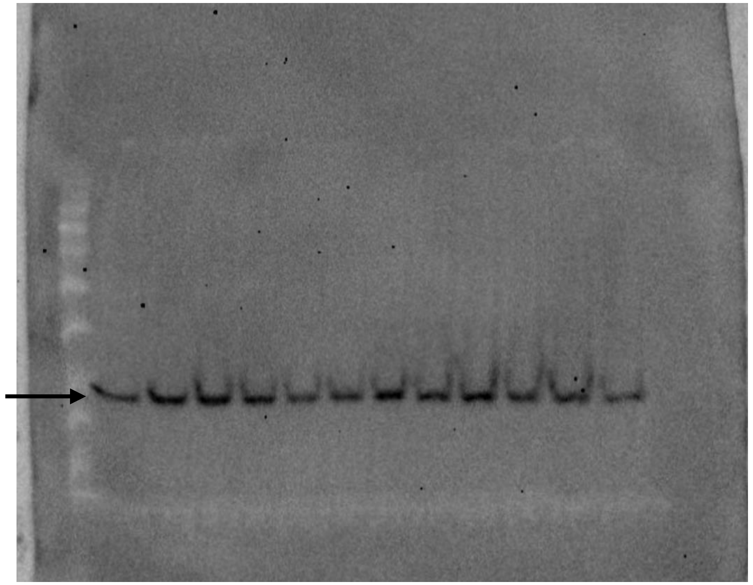

GAPDH (37-50 kDa)
Saline Treatment

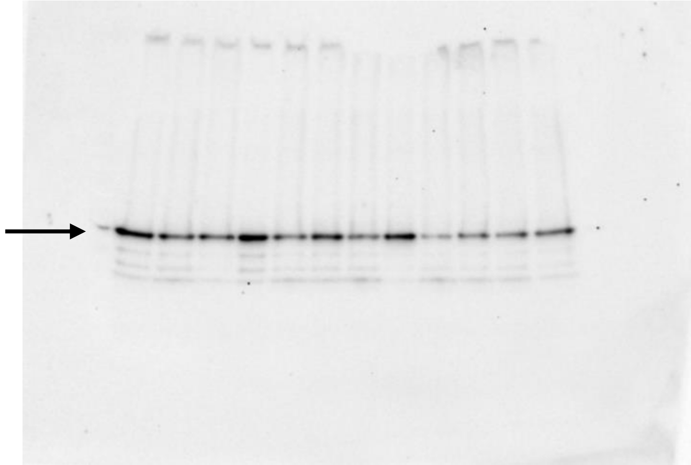

Supplement: Supplementary file 5 [file Datasheet10.pdf]
