# Supplementary material for: Behavioral and biochemical changes associated with the analgesic effects of (2R,6R)-hydroxynorketamine alone and in combination with meloxicam following disk puncture in mice
Source: Front Pain Res (Lausanne). 2025 Jun 12;6:1574474. doi: 10.3389/fpain.2025.1574474 (PMC12203739; doi:10.3389/fpain.2025.1574474)

Supplemental figure 2
Full WB images Dorsal RootGanglia

Behavioral and Biochemical Changes Associated with the Analgesic Effects of (2R,6R)-Hydroxynorketamine Alone and in Combination with Meloxicam Following Disk Puncture in Mice

DRG: TRPA1

Female

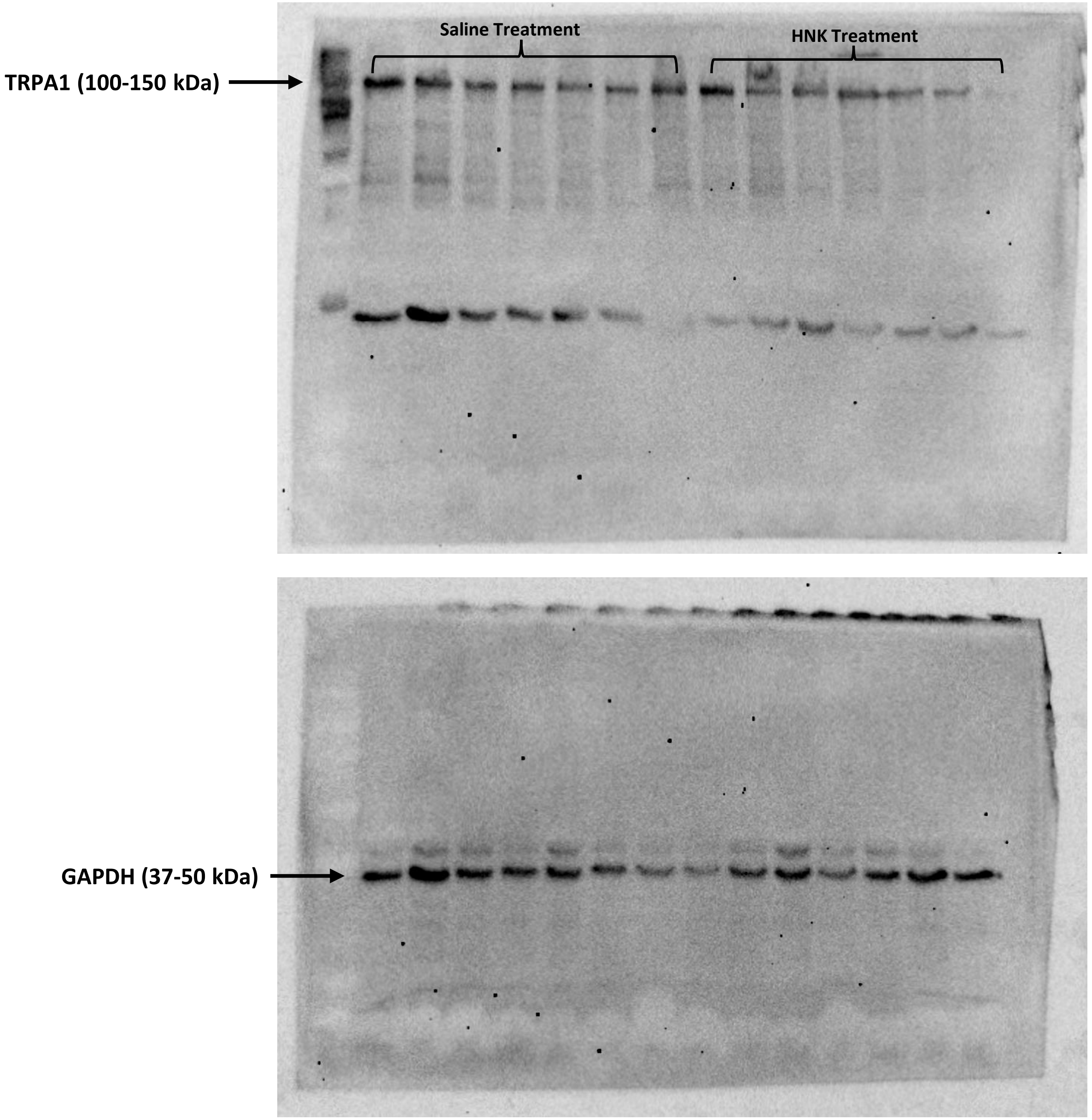

Male

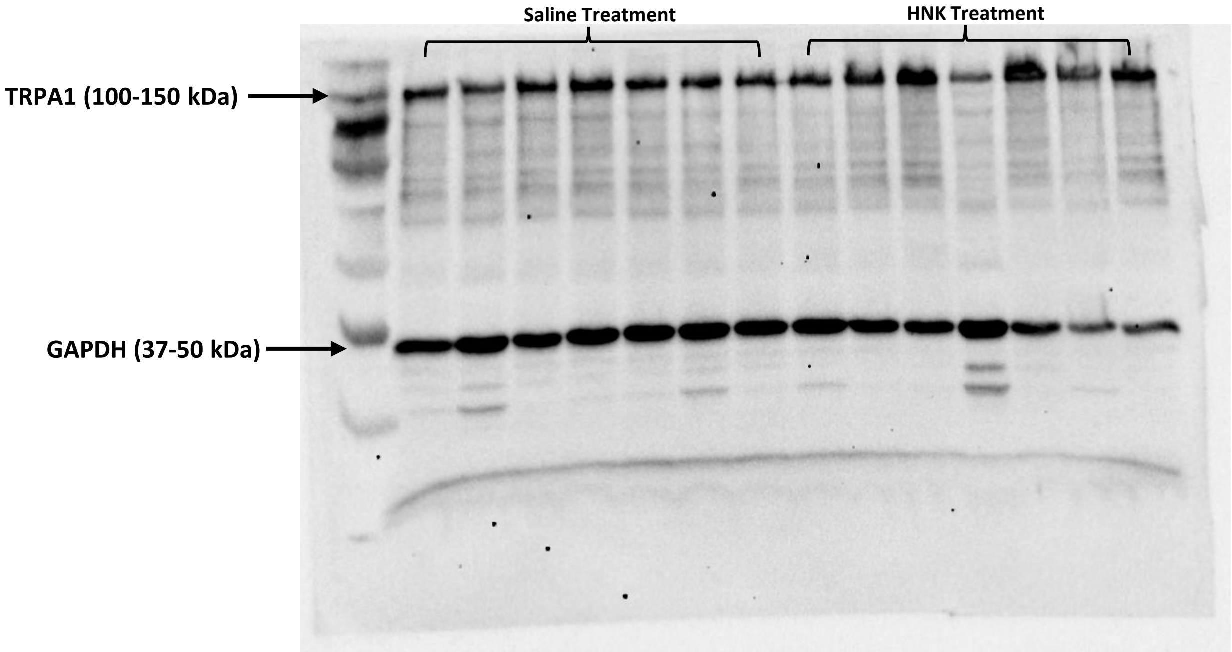

DRG: TRKB

Female

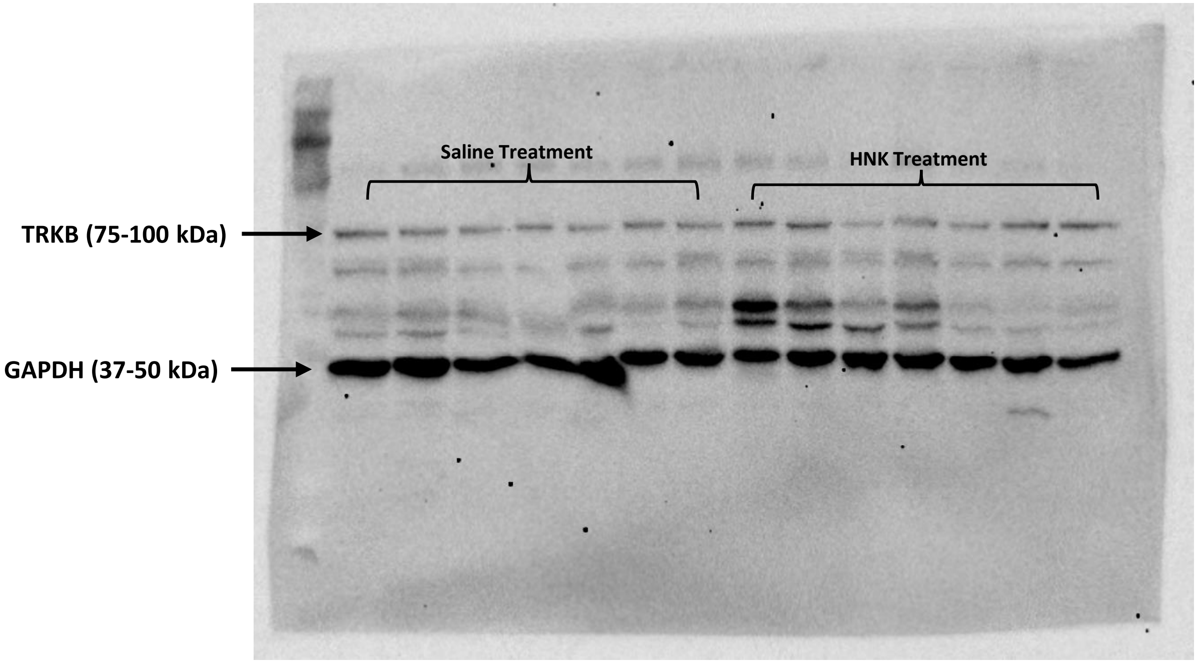

Male

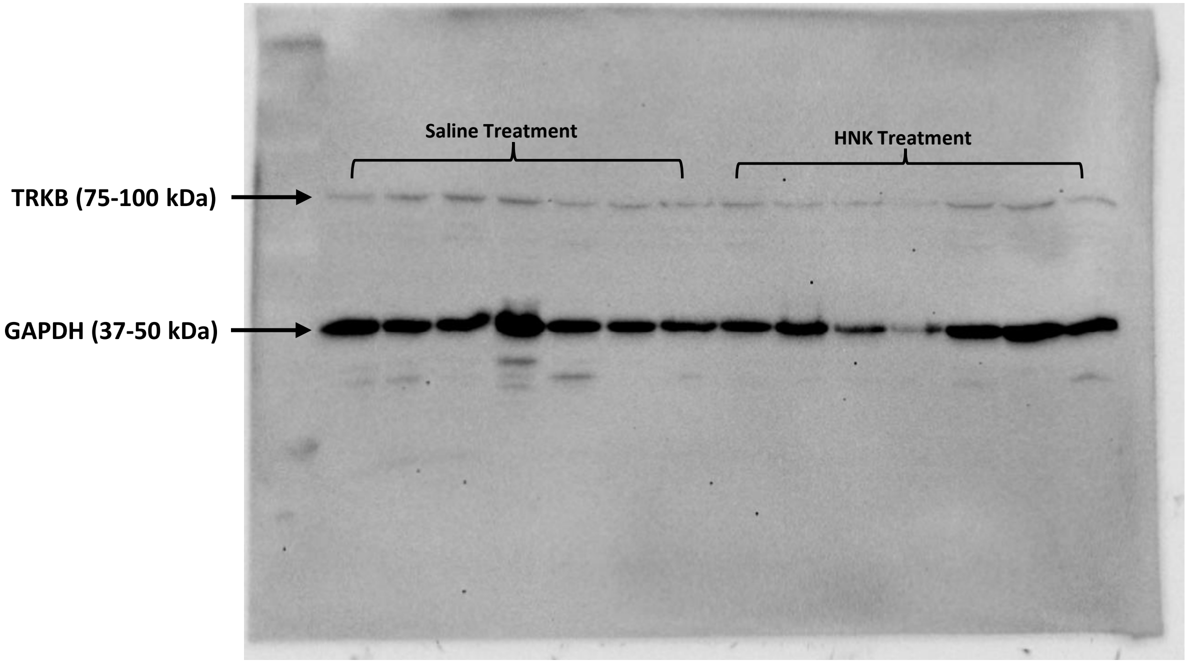

DRG: CXCR4

Female

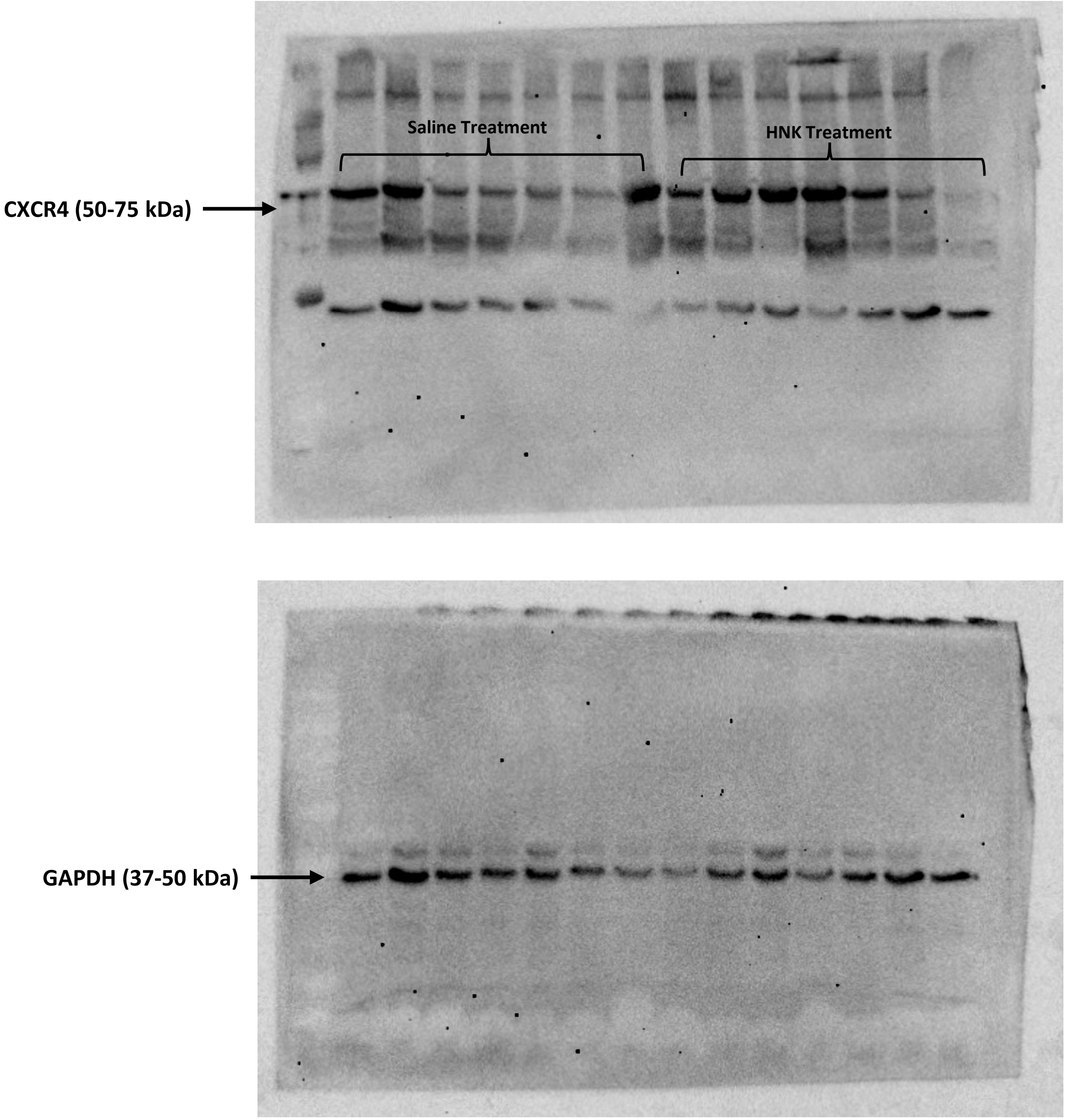

Male

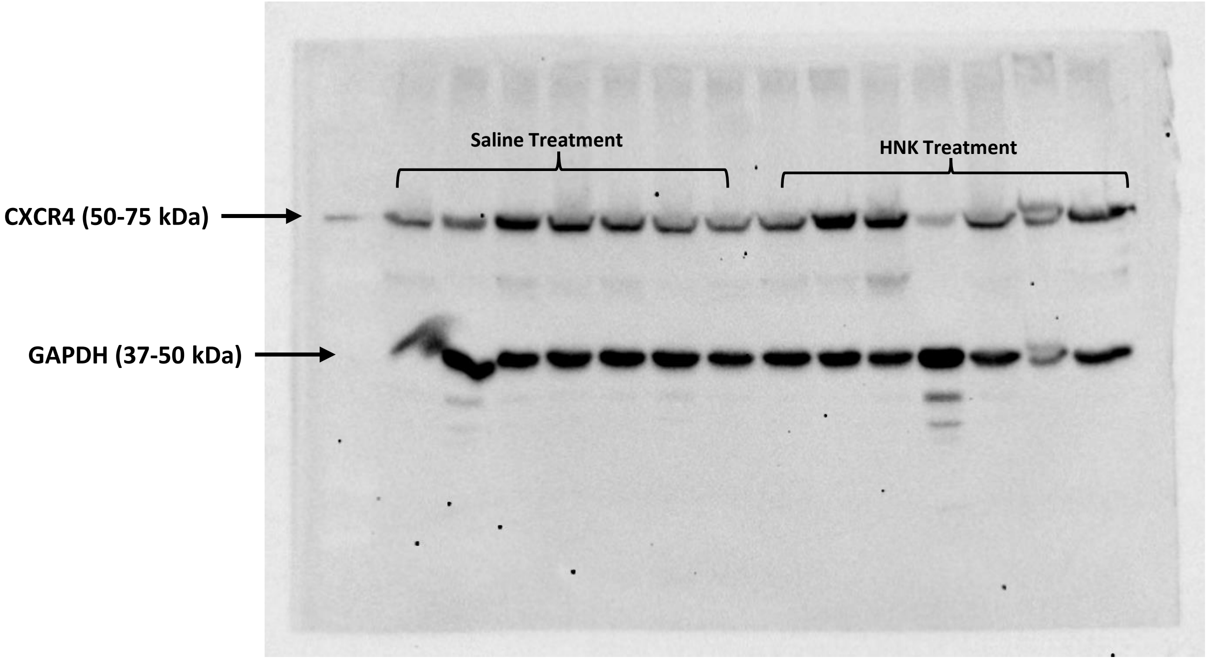

DRG: pERK

Female

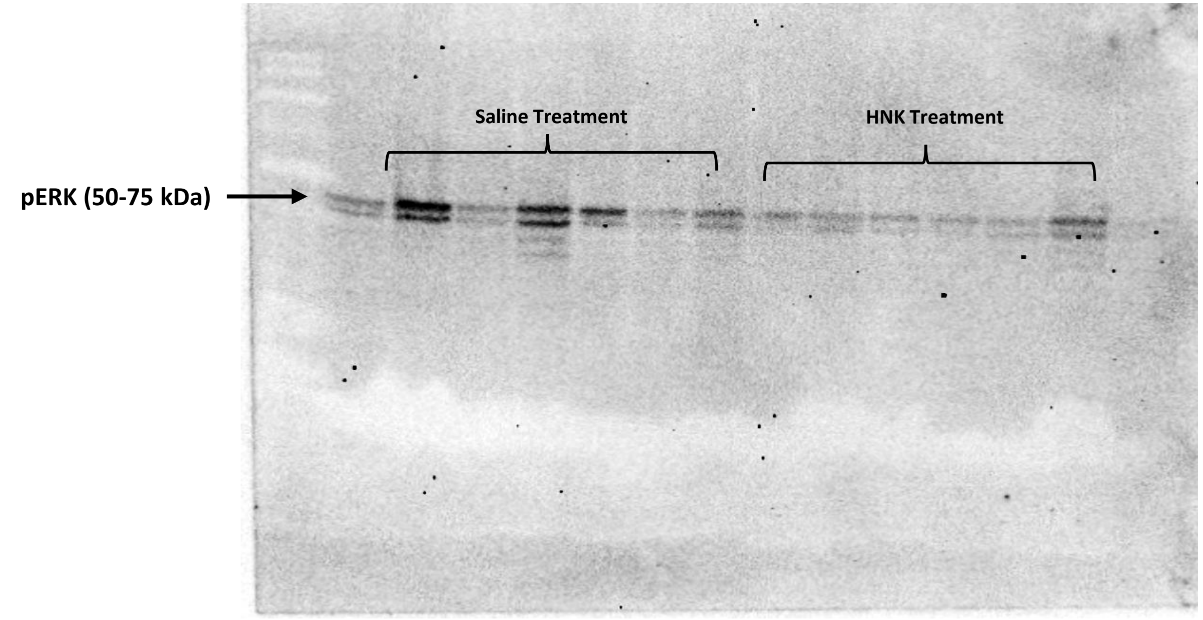

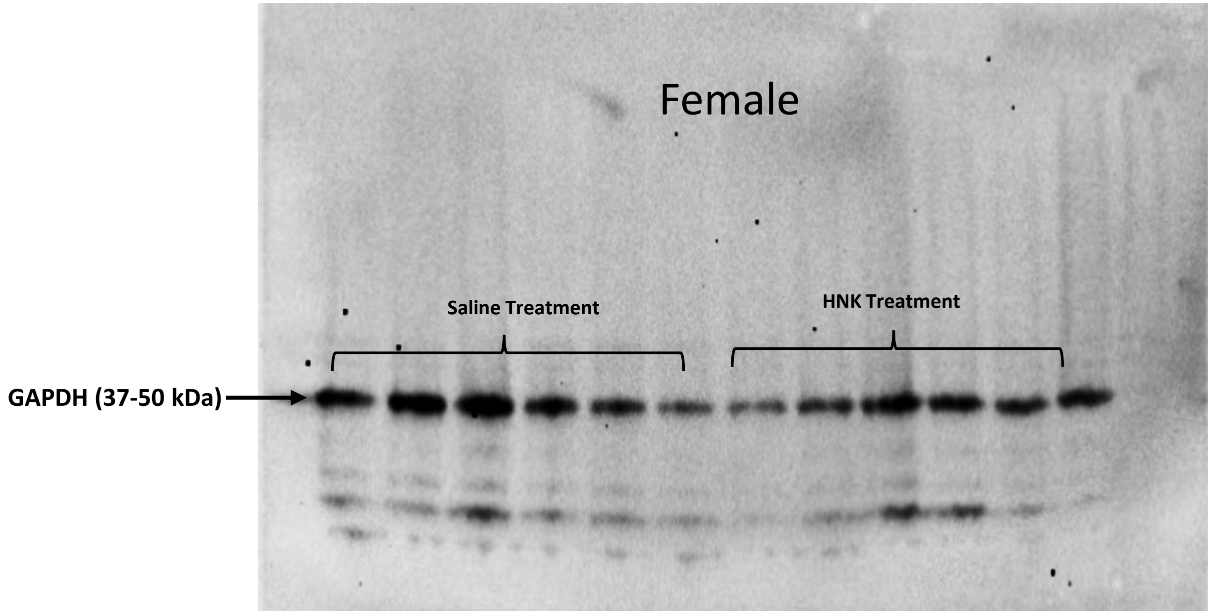

Male

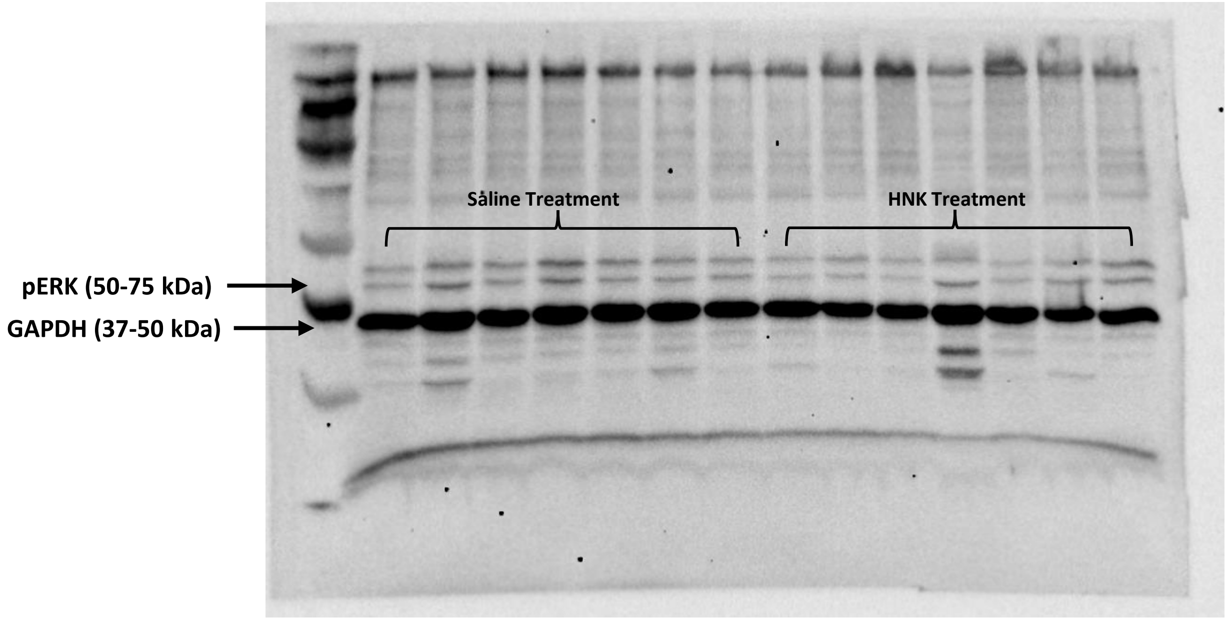

DRG: BDNF

Female

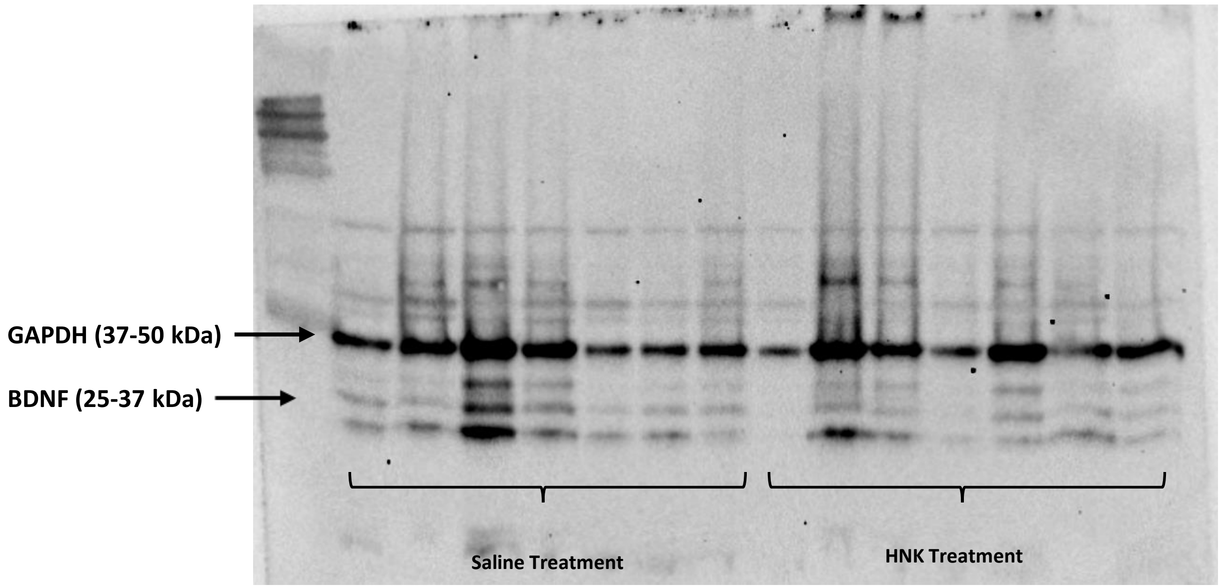

Male

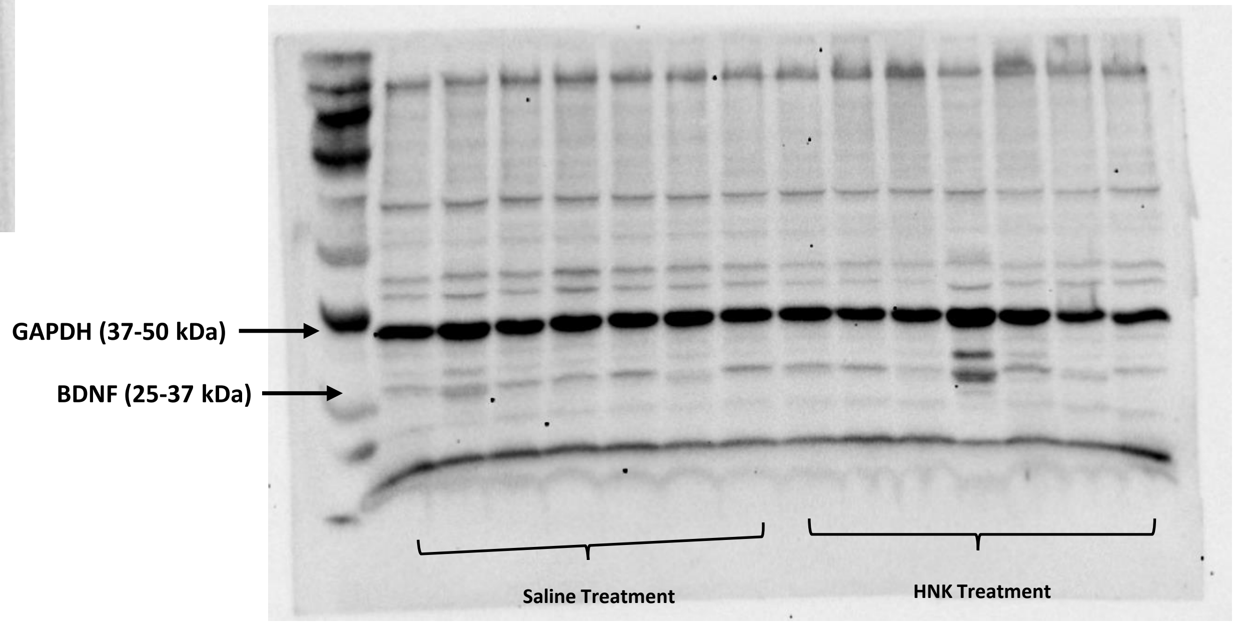

DRG: EIF4E

Female

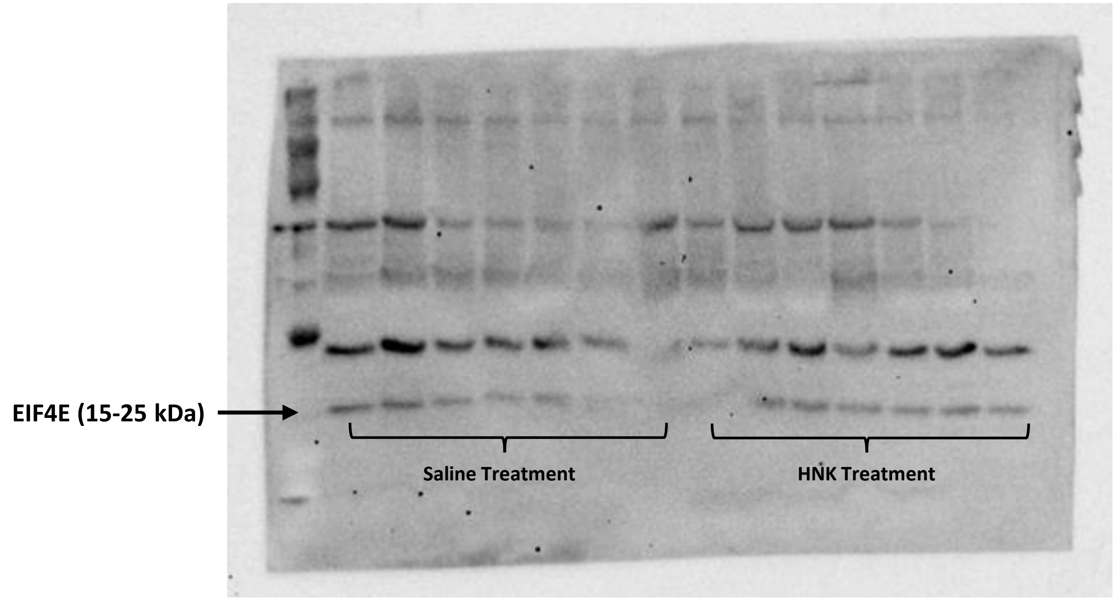

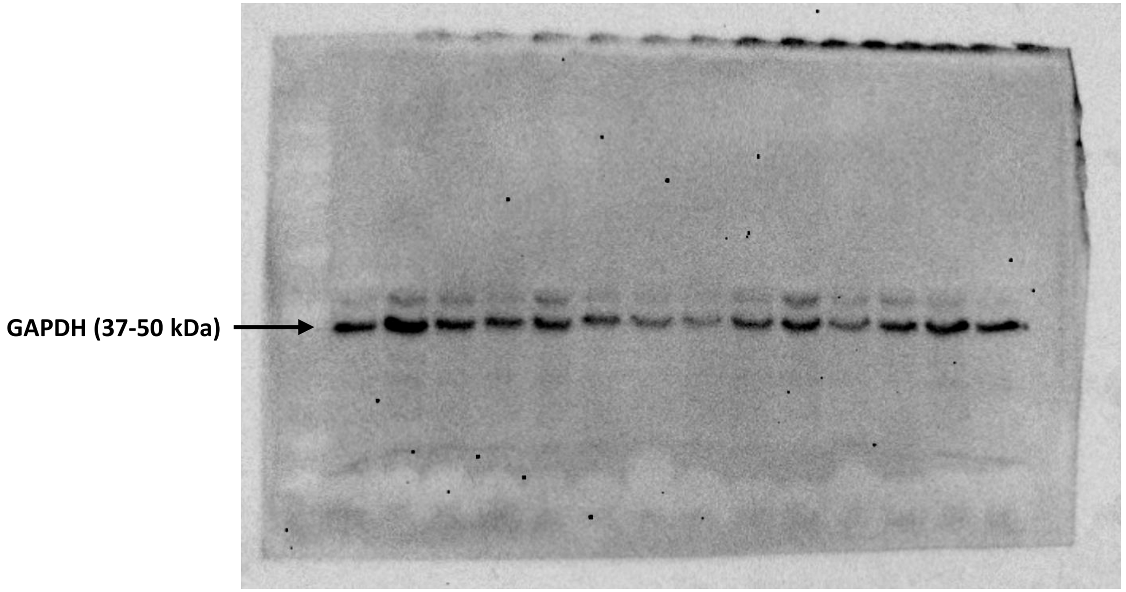

Male

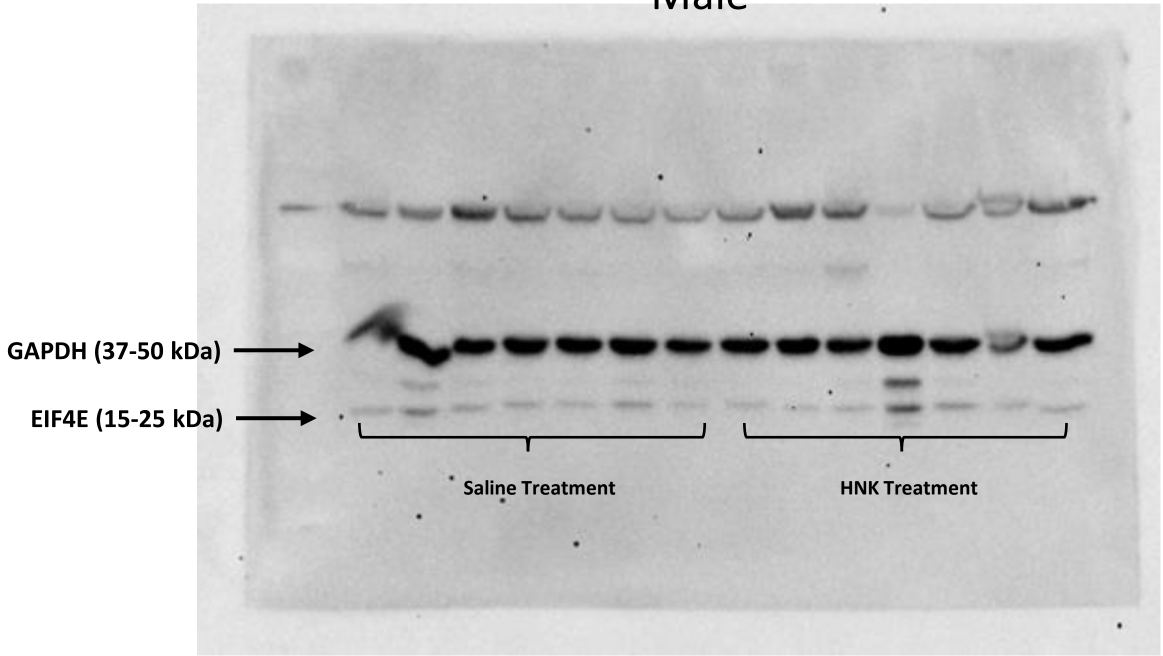

Supplement: Supplementary file 6 [file Datasheet11.pdf]
