# Supplementary figures and images for: Behavioral and biochemical changes associated with the analgesic effects of (2R,6R)-hydroxynorketamine alone and in combination with meloxicam following disk puncture in mice
Source: Front Pain Res (Lausanne). 2025 Jun 12;6:1574474. doi: 10.3389/fpain.2025.1574474 (PMC12203739; doi:10.3389/fpain.2025.1574474)

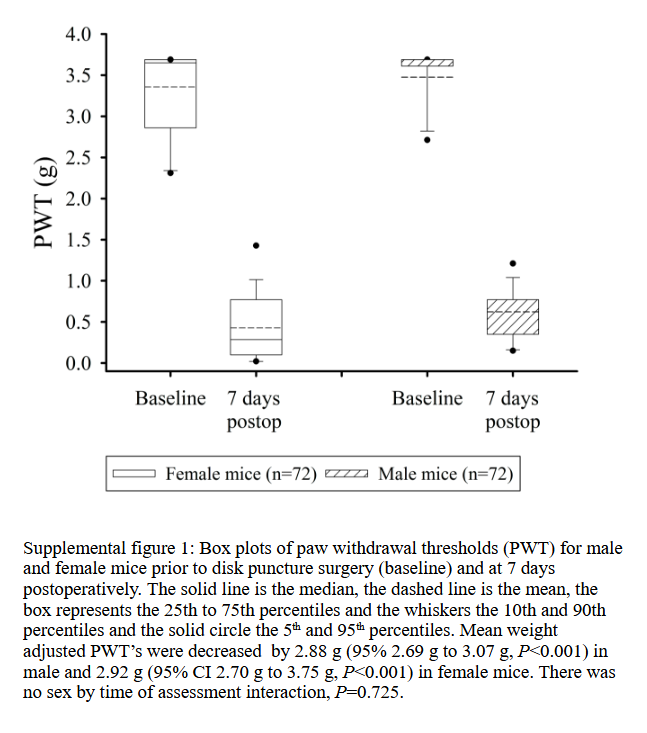

Supplement: Supplementary file 7 [file Image5.tiff]

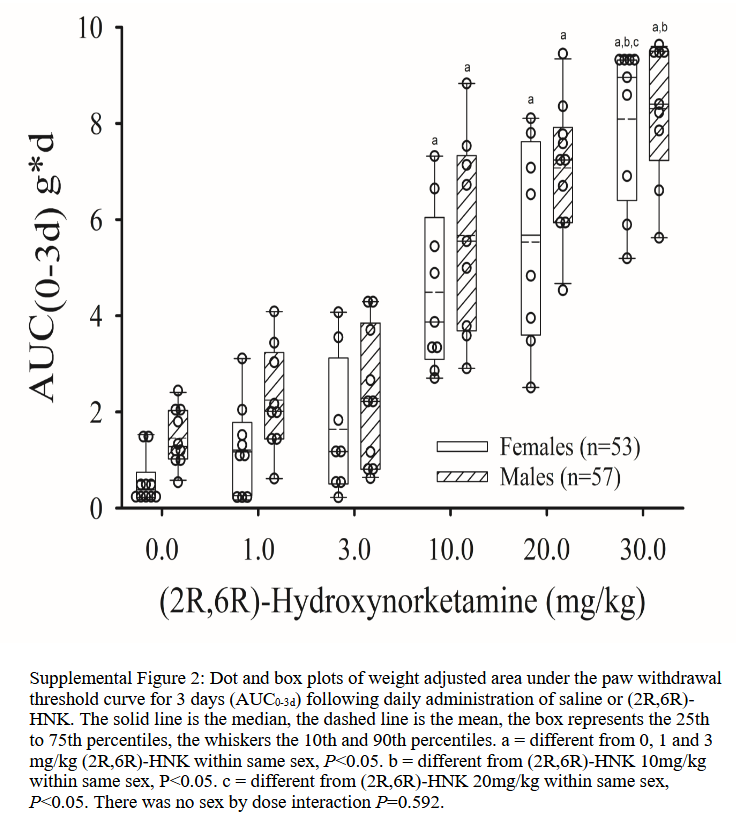

Supplement: Supplementary file 8 [file Image6.tiff]

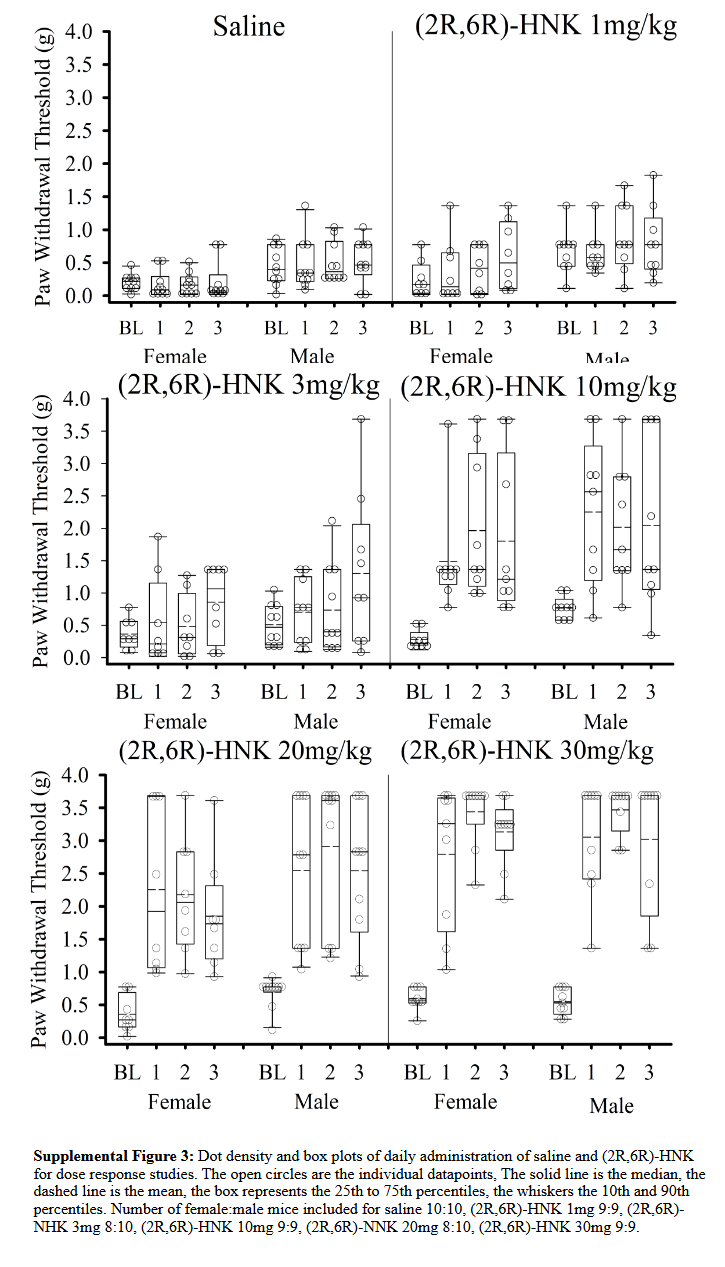

Supplement: Supplementary file 9 [file Image7.tiff]

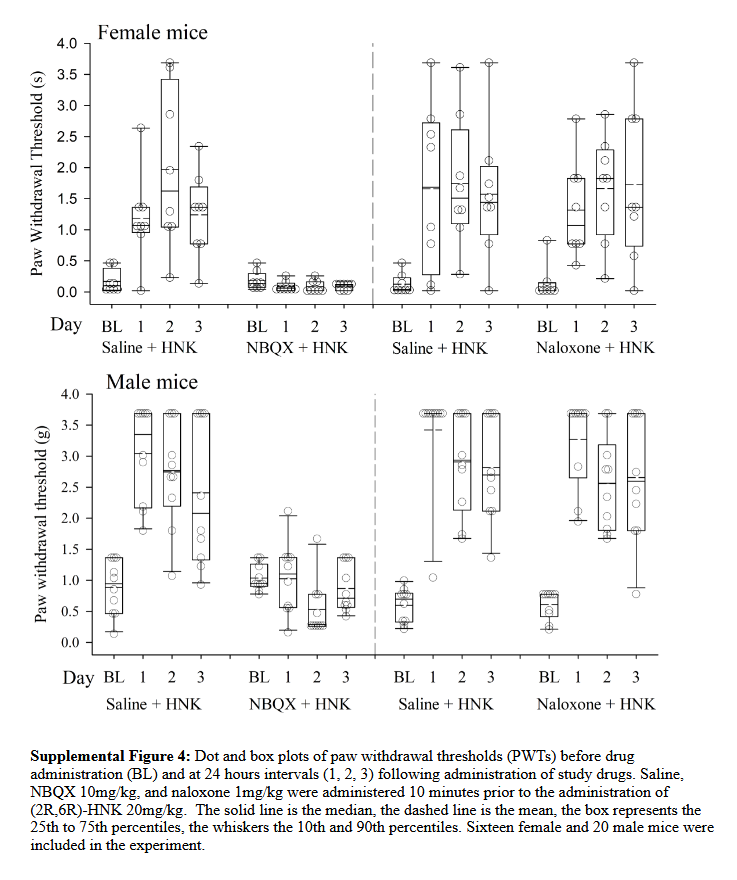

Supplement: Supplementary file 10 [file Image8.tiff]

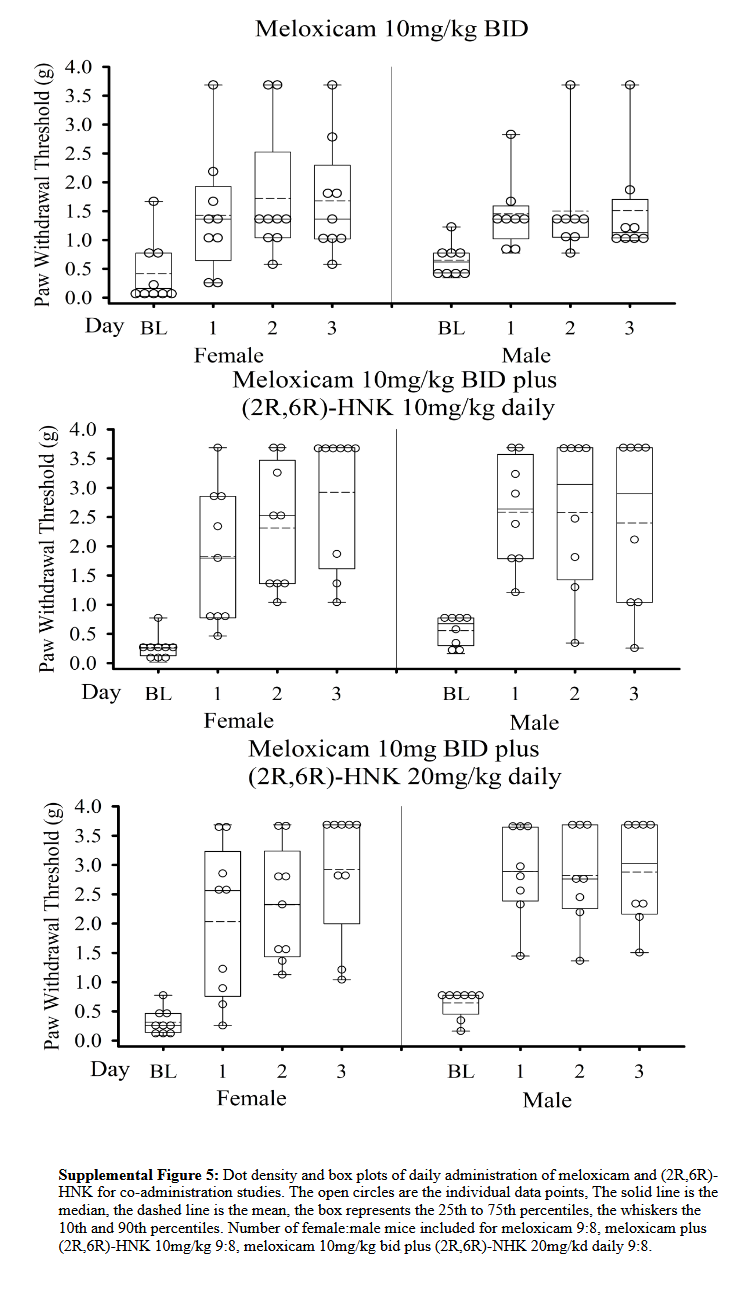

Supplement: Supplementary file 11 [file Image9.tiff]
